# Genetic insight into Birt–Hogg–Dubé syndrome in Indian patients reveals novel mutations at *FLCN*

**DOI:** 10.1186/s13023-022-02326-5

**Published:** 2022-04-27

**Authors:** Anindita Ray, Esita Chattopadhyay, Richa Singh, Saurabh Ghosh, Arnab Bera, Mridul Sarma, Mahavir Munot, Unnati Desai, Sujeet Rajan, Pralhad Prabhudesai, Ashish K. Prakash, Sushmita Roy Chowdhury, Niladri Bhowmick, Raja Dhar, Zarir F. Udwadia, Atin Dey, Subhra Mitra, Jyotsna M. Joshi, Arindam Maitra, Bidyut Roy

**Affiliations:** 1 Human Genetics Unit, Indian Statistical Institute, Kolkata, India; 2 Department of Pulmonary Medicine, RG Kar Medical College and Hospital, Kolkata, India; 3 Respiratory Medicine and Critical Care, Medica Superspeciality Hospital, Kolkata, India; 4 Department of Chest Medicine, Calcutta National Medical College, Kolkata, India; 5 Narayana Superspeciality Hospital, Guwahati, India; 6 Department of Pulmonary Medicine, TNMC and BYL Nair Hospital, Mumbai, India; 7 Department of Chest Medicine, Bombay Hospital Institute of Medical Sciences, Mumbai, India; 8 Lilavati Hospital and Research Centre, Mumbai, India; 9 Department of Respiratory and Sleep Medicine, Medanta- The Medicity, Gurgram, India; 10 Apollo Hospital Kolkata, Pulmonology, India; 11 Fortis Hospital Kolkata, Pulmonology, India; 12 Department of General Medicine, IPGMER&SSKM Hospital, Kolkata, India; 13 CMRI, C K Birla Group of Hospitals, Kolkata, India; 14 P.D. Hinduja Hospital and Research Center, Mumbai, India; 15 National Institute of Biomedical Genomics, Kalyani, India; 16 Department of Molecular Cell Biology, Weizmann Institute of Science, Rehovot, Israel; 17 Pathology and Laboratory Medicine, Weill Cornell Medicine, New York, NY USA

**Keywords:** *FLCN* mutations, BHDS, Primary spontaneous pneumothorax, Family-based association, Molecular docking, Indian patients

## Abstract

**Background:**

Birt-Hogg-Dubé syndrome (BHDS) is a rare monogenic condition mostly associated with germline mutations at *FLCN*. It is characterized by either one or more manifestations of primary spontaneous pneumothorax (PSP), skin fibrofolliculomas and renal carcinoma (chromophobe). Here, we comprehensively studied the mutational background of 31 clinically diagnosed BHDS patients and their 74 asymptomatic related members from 15 Indian families.

**Results:**

Targeted amplicon next-generation sequencing (NGS) and Sanger sequencing of *FLCN* in patients and asymptomatic members revealed a total of 76 variants. Among these variants, six different types of pathogenic *FLCN* mutations were detected in 26 patients and some asymptomatic family members. Two of the variants were novel mutations: an 11-nucleotide deletion (*c.1150_1160delGTCCAGTCAGC*) and a splice acceptor mutation (*c.1301-1G* > *A*). Two variants were Clinvar reported pathogenic mutations: a stop-gain (*c.634C* > *T*) and a 4-nucleotide duplication (*c.1329_1332dupAGCC*). Two known variants were: hotspot deletion (*c.1285delC*) and a splice donor mutation (*c.1300* + *1G* > *A*). *FLCN* mutations could not be detected in patients and asymptomatic members from 5 families. All these mutations greatly affected the protein stability and *FLCN-FNIP2* interaction as observed by molecular docking method. Family-based association study inferred pathogenic *FLCN* mutations are significantly associated with BHDS.

**Conclusion:**

Six pathogenic *FLCN* mutations were detected in patients from 10 families out of 15 families in the cohort. Therefore, genetic screening is necessary to validate the clinical diagnosis. The pathogenic mutations at *FLCN* affects the protein–protein interaction, which plays key roles in various metabolic pathways. Since, pathogenic mutations could not be detected in exonic regions of *FLCN* in 5 families, whole genome sequencing is necessary to detect all mutations at *FLCN* and/or any undescribed gene/s that may also be implicated in BHDS.

**Supplementary Information:**

The online version contains supplementary material available at 10.1186/s13023-022-02326-5.

## Background

Birt-Hogg-Dubé syndrome (BHDS) [MIM: 135150]) is a rare inherited condition, first described in 1977 with skin fibrofolliculomas on the forehead, neck and upper torso of patients [1]. Presence of familial primary spontaneous pneumothorax (PSP; [MIM: 173600]) and/or lung cysts, and chromophobe or oncocytic renal cell carcinoma are two other manifestations in BHDS [2, 3]. It follows autosomal dominant pattern of inheritance with incomplete penetrance [4] and is known to be monogenic, associated with germline mutations at *Folliculin* (*FLCN*; NM_144997.5) located at *17p11.2* [5]. More than 290 pathogenic germline mutations at *FLCN* have been reported in BHDS [6]. Among these, a hotspot protein truncating mutation in a hypervariable poly-*C* tract (*C*_8_) in exon 11 (*c.1285)* has been reported in different populations [7, 8]*.* BHDS chromophobe renal tumours exhibit loss of heterozygosity (LOH), suggesting a tumour suppression role of *FLCN* in kidney [9]. Haploinsufficiency at *FLCN* has also been reported in BHDS manifestations [10, 11]. FLCN protein is similar to DENN domain proteins, though its exact function is unknown [12]. It interacts via its carboxy-terminal (C-terminal) with two proteins—Folliculin interacting protein 1 and 2 (FNIP1 and FNIP2) [5]. These complexes play important roles in major metabolic pathways such as modulation of mTOR pathway [13], AMPK activation [14, 15], PGC1-α regulation, mitochondrial biogenesis [16], GAP dependant mTORC1 activation [17], HIFα transcription [18], cell–cell adhesion [19], membrane trafficking [20], autophagy [21], ciliogenesis [22], and cell cycle progression [23].

Skin fibrofolliculomas and pathogenic *FLCN* mutations are two major diagnostic criteria for BHDS, while lung and kidney phenotypes, and presence of first degree family history are known to be minor criteria [24]. However, these manifestations could be population-specific, as skin fibrofolliculomas are not prevalent in East Asian cohorts [25]. A few other similar conditions like Homocystinuria, alpha-1 antitrypsin deficiency, vascular Ehlers-Danlos syndrome, Lymphangioleiomyomatosis (LAMS) may have overlapping pulmonary phenotypes like BHDS, thus confounding disease diagnosis [26].

Studies of more than 600 BHDS families have been reported world-wide with majority of them from the USA and Europe, fewer from Asia (mostly from East Asia) with only one from India [27]. Here, we have comprehensively profiled germline mutations in BHDS patients and related family members from 15 Indian families and predicted molecular mechanisms for disease phenotype.

## Methods

### Ethics statement

The study was approved by the "Review committee for protection of research risk to humans, Indian Statistical Institute, 2015". Written informed consent from all adult participants and legal guardians/parents for minors was obtained for the research study using blood samples and subsequent publication of the results.

### Clinical characterization of study population

Patient IDs were assigned anonymously for families, patients and asymptomatic members. We enrolled 31 clinically diagnosed BHDS patients, during 2015–2019, with PSP or BHDS-specific lung cysts along with skin and/or renal manifestations, with/without a positive family history and their 74 asymptomatic family members (Additional file 1: Table S1). Recruitment was done with the help of clinicians from different hospitals in India. Clinical phenotype of each patient was also determined using human phenotype ontology terms (HPO), a web-based tool, Phenomizer [28, 29]. It evaluates patient-specific HPO terms and assigns a p-value to the suspected disease of the patient, based on their ranks through Benjamini–Hochberg multiple correction test.

### Detection of inherited variants at *FLCN* by different sequencing methods

#### Targeted amplicon next-generation sequencing (NGS)

Initially, genomic DNA from blood of 20 patients and 15 related asymptomatic members from 11 families were isolated for targeted amplicon NGS (Additional file 1: Table S2). Patient F1-1 was included as a positive control, as mutation at *FLCN* was previously determined by us [27]. All patients and related asymptomatic family-members were not included for NGS study to minimize sequencing manual errors and logistic problems. The 24 kb *FLCN*, including UTRs, exons and flanking introns (Additional file 1: Table S3a) was amplified by long PCR. Exon 6 and its flanking 2.8 kb intronic region could not be amplified due to technical limitations but were studied by Sanger sequencing method. Targeted amplicon generation and equimolar pooling were performed (Additional file 2: Methods) before library preparation, which was done using Nextera XT Library Preparation kit (Illumina Inc.). Paired-end 100 bp sequencing was performed in Illumina HiSeq 2500 platform. Adaptor trimmed sequence reads were mapped to the human reference genome build *(hg38)* using BWA-mem. Standard pipelines were followed for quality filtering and metrics assessment. Germline mutations were called by three variant callers such as Haplotype Caller, STRELKA, VarScan2 (Additional file 2: Methods) [30–36].

#### Validation of pathogenic variants and detection of inherited variants in remaining samples

Bidirectional Sanger sequencing of all exons (Additional file 1: Table S3b) was performed for members of 4 more families; F12, F13, F14 and F15 (9 patients and 20 asymptomatic family members) to detect *FLCN* mutations. Pathogenic variants (discovered from NGS) were also validated in all individuals from 11 families (Additional file: Table S2). BioEdit and *in-silico* tools were used for sequence alignment and variant analysis [37, 38].

### e-QTL analysis for expression and population frequencies of *FLCN* variants

Effect of non-coding germline variants on *FLCN* expression (if any) were examined using computed expression quantitative trait loci (e-QTL) data from GTEx [39]. Since BHDS is a rare disease, population-specific alternate allele frequencies of the non-coding variants were checked in South Asian/Indian population (gnomAD, GenomeAsia100K database) [40, 41].

### Pedigree disequilibrium test (PDT) for association study

Pathogenic variants and regulatory SNPs at *FLCN* were tested for association with BHDS in families by PDT. It is based on a test statistic, *T*, which, for a one-tailed test with 5% significance, is considered as significant if values of* T* are ≥ 1.64 (Additional file 2: Methods) [42].

### Homology modelling and molecular docking

The cryo-EM structure of FLCN-FNIP2-Rag-Ragulator complex (pdb code: *6ulg*) was taken as template for modelling wild-type (wt)/mutant FLCN, wt-FNIP2, wt-Ras-related GTP-binding protein A and protein C (RRAGA and RRAGC) monomers using SWISS-MODEL web server. (Additional file 2: Methods). Monomer-models were visualized at Pymol and validated with PROCHECK [43]. Two sets of molecular docking were performed for wild-type and four mutant monomers of *FLCN*, each with (i) wt-FNIP2, wt-RRAGA, wt-RRAGC together (4-protein complex), and (ii) wt-FNIP2 (2-protein complex). HADDOCK 2.4 web server *Guru Interface* was used for macromolecular docking with default parameters for running the program and subsequent analysis (Additional file 2: Methods) [44].

### Copy number variation of *FLCN*

Read count normalization of *FLCN* amplicons generated by NGS of 35 samples was performed by Seqmonk (Additional file 2: Methods) to get an initial indication of any *FLCN* copy number difference between patients and asymptomatic members in different families. Subsequently, to validate NGS copy number variation data, Taqman copy number assay was performed in 4 families—F3, F4, F9, F15 (Additional file 1: Table S4) and 23 unrelated healthy controls (data not shown). Taqman copy number assays for exon 4 (Hs01200751_cn), exon 8 (Hs01889931_cn), exon 13 (Hs01203178_cn) of *FLCN* and *RNase P* (as reference) were performed in real-time PCR instrument. Ct values of *FLCN* and *RNase P* were used to determine copy numbers of *FLCN* (Additional file 2: Methods) following statistical analyses in SPSS.

## Results

### Demography and clinical manifestations

Thirty out of 31 clinically diagnosed BHDS patients presented BHDS lung phenotype, with 10 patients also manifesting skin fibrofolliculomas, with/without a positive family history. Patient, F7-49, only presented skin fibrofolliculomas. Three patients also presented chromophobe renal cancer (Additional file 1: Table S5). The male to female ratio was 58% and 41.9% in patients and asymptomatic members, respectively. Age ranged from 18 to 87 years in patients and 7 to 88 years in asymptomatic members. The number of smokers were observed to be more prevalent in patients than asymptomatic members (Table 1).

**Table 1:** Demography and clinical manifestations of patients (n = 31) and asymptomatic members (n = 74)

Patients (n = 31)	Demography
Mean age ± SD (range in years)	49 ± 15.4, (18–87)
Males	58% (n = 18)
Females	41.9% (n = 13)
Smoking habit (> 10 years)	22% (n = 7)
History of tuberculosis	16% (n = 5)

BHDS diagnostic criteria	Number of patients*
Patients with lung cysts or PSP	96% (n = 30)
Patients with skin fibrofolliculomas	35% (n = 11)
Patients with renal cysts/carcinoma (chromophobe)	9.6% (n = 3)
Patients having 1^st^ degree relative with similar respiratory phenotype	64% (n = 20)

Asymptomatic family members	n = 74
Mean age ± SD (range in years)	36 ± 19.8 (7–88)
Males	58% (n = 43)
Females	41.8% (n = 31)
Smoking habit (> 10 years)	1.3% (n = 1)
History of tuberculosis	6.75% (n = 5)
*BHDS diagnostic criterion (except respiratory or dermal manifestation)*	
Asymptomatics with renal cysts/carcinoma**	2.7% (n = 2)

^*^Some of the patients had more than one phenotype

^**^Two individuals from family F7 only presented renal cell carcinoma, but were not clinically evaluated as BHDS. They were third degree relatives of the index patient, therefore they were considered as asymptomatic related members of the index patient

### Clinical characterization of patients

Clinical histories of the patients were examined by clinicians from different hospitals. Phenotype ontology analysis revealed 17 HPO terms using Phenomizer (Additional file 3: Figure S1), which assigned PSP and BHDS to 23 of 31 patients, with significant p-values of ≤ 0.05 (Additional file 1: Table S6 and Additional file 3: Fig. S2). Patient ontology for three patients of family F9 did not qualify for PSP or BHDS. Inconclusive results were obtained for patients, F5–26 and F5–28 (family F5), and F15-99 (family F15), however index patients of both families were significantly assigned with PSP.

### Germline mutations at *FLCN*

**Fig. 1:**
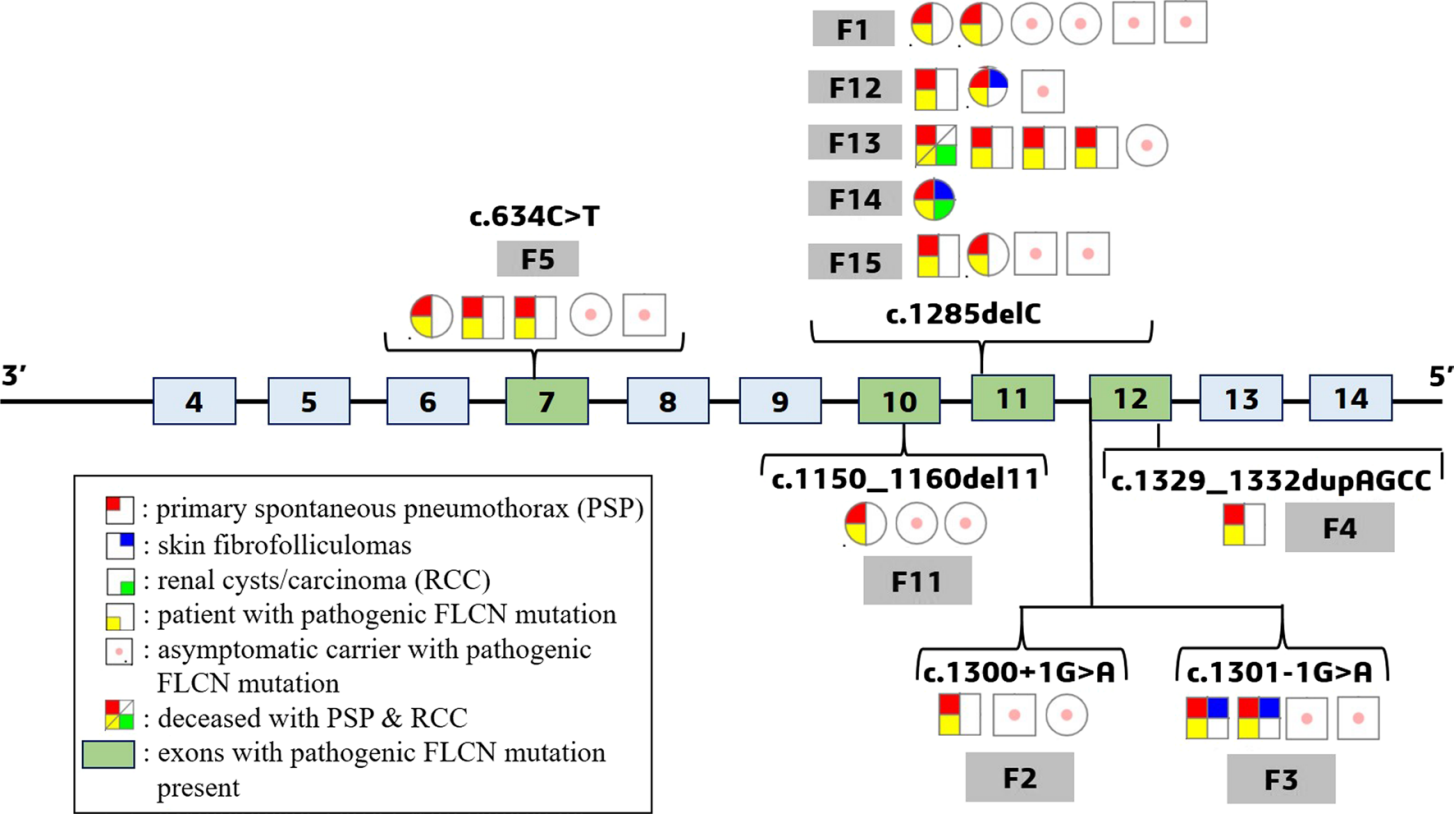
Pathogenic mutations at *FLCN* found in patients and asymptomatic members along with their phenotypes. Light blue boxes represent exons and green boxes are exons with pathogenic mutations in *FLCN*. Most pathogenic mutations were found between exons 10–13. Circles indicate females, squares males. Family numbers are denoted in grey boxes with patients and asymptomatic members harboring pathogenic *FLCN* mutations. Patient phenotypes are also indicated in red, blue and green colors in individual symbols, and asymptomatic carriers are denoted with a pink dot

An average of 7 million reads per sample were obtained from targeted amplicon NGS data (Additional file 1: Table S7), and after various quality filters, it revealed a total of 412 variants (Additional file 3: Fig. S3). Variants from homo-polymeric regions (> 9) were removed to obtain a total of 76 variants. Among these variants; 4 exonic and 2 splice region mutations were found to be pathogenic. Sanger sequencing of *FLCN* exons validated these 6 pathogenic mutations detected in NGS with 100% concordance (Additional file 3: Figs. S4-1, S4-2, S4-3, S4-4, S4-5 and S4-6). Pathogenic *FLCN* mutations were detected in 10 out of 15 families recruited in the study. These pathogenic variants were found in 19 of 31 patients and 16 of 74 related asymptomatic members in 10 families (Fig. 1 and Table 2). All 19 patients with pathogenic *FLCN* mutations presented PSP and/or BHDS lung cysts. Patient F14-95 harboured all three BHDS manifestations (PSP, skin fibrofolliculomas and renal cysts). Two more patients (F3-13, F12-77) had skin fibrofolliculomas, and patient F13-82 had chromophobe renal cell carcinoma. Remaining 70 variants were found in UTRs and introns of *FLCN* (Additional file 1: Tables S8a and S8b) and among these; 13 and 12 variants were reported as Clinvar 'benign' and non-pathogenic, respectively. Among the 6 pathogenic variants (Fig. 1), two were heterozygous novel mutations: a frame-shift deletion of 11 nucleotides, *c.1150_1160delGTCCAGTCAGC (c.1150_1160del11)* in exon 10 in family F11, and a splice acceptor mutation, *c.1301-1G* > *A* in intron–exon boundary of exon 12 in family F3. A stop-gain mutation, *c.634C* > *T* in exon 7, and a frame-shift duplication of 4 nucleotides, *c.1329_1332dupAGCC* in exon 12, were found in family F5 and F4, respectively, which were Clinvar reported 'pathogenic' heterozygous mutation. The hotspot heterozygous deletion mutation, *c.1285delC* in exon 11 was found in 5 families (F1, F12, F13, F14, F15). A reported heterozygous splice donor mutation, *c.1300* + *1G* > *A* was found in exon–intron boundaries of exon 12 in family F2 (Additional file 3: Figs. S4-1, S4-2, S4-3, S4-4, S4-5 and S4-6).

**Table 2:** Pathogenic variants at *FLCN* in 19 patients and 16 related asymptomatic members

COORD	SNP ID	HGVS	Consequence	Exon	Annotation	Ref	Alt	Patients	Asymptomatic members	Reports
17,222,646	*rs558699420*	*c.634C* > *T*	*p.Gln212Ter*	7	Stop-gain	*C*	*T*	F5–25, F5–26, F5–27	F5–24, F5–32	Clinvar pathogenic
17,217,085–17,217,095	–	c.1150_1160del GTCCAGTCAGC*	*p.Val384Phefs*	10	Deletion	*GTCCAGTCAGC*	*GTCCAGTCAGC/–*	F11–70	F11–73, F11–74	Novel
17,216,395	*rs80338682*	*c.1285delC*	*p.His429Thrfs*	11	Deletion	*C*	*C/*–	F1–1, F1–2, F12–77, F12–78, F13–82 to F13–85, F14–95, F15–99, F15–101	F1–4 to F1–7, F12–80, F13–89, F15–105, F15–106	Reported (hotspot)
17,215,284	–	*c.1329_1332dupAGCC*	*p.Ala445Serfs*	12	Duplication	*AGCC*	–*/AGCC*	F4–18	None	Clinvar pathogenic
17,216,379	*rs879255676*	*c.1300* + *1G* > *A*	Splice donor	11–12	Splice region	*C*	*T*	F2–9	F2–10, F2–12	Reported
17,215,317	–	*c.1301−1G* > A*	Splice acceptor	11–12	Splice region	*C*	*T*	F3–13, F3–14	F3–16, F3–17	Novel

^*^Novel mutations

*COORD* Genomic coordinates, *Ref* reference allele, *Alt* alternate allele

### Expression of *FLCN*: e-QTL study from GTex database

Low *FLCN* expression is reported in tissues (especially in kidney) of BHDS patients [39, 45]. Therefore, we checked whether non-coding variants also affect *FLCN* expression. We detected 70 non-coding variants and found 12 SNPs (Table 3) common with e-QTL data of *FLCN* expression (for lung and skin tissues—Additional file 1: Table S9a), with alternate allele frequencies ≤ 15% in South Asian population (Additional file 1: Table S9b). These SNPs may affect *FLCN* expression and among these, alternate allele genotypes *(CT/TT & AG/GG)* of two SNPs: *rs41345949 (C* > *T)* and *rs41525346 (A* > *G),* were found to be more frequent in patients compared to their asymptomatic family members. The *rs41345949*, although Clinvar benign, is a highly conserved regulatory SNP with an alternate allele frequency of < 3% in Indian population. Interestingly, the *TT* and/or *CT* genotypes were found in patients of families without any *FLCN* pathogenic mutations (families F6 and F7). They were also found in patients, F15-99 and F15-101 (genotype *TT*), who also harbour *c.1285delC FLCN* mutation, and patient F4-18 (genotype *CT*), also harboring *c.1329_1332dupAGCC* mutation. The *rs41525346* is an intronic SNP, highly conserved, and has a distal enhancer-like signature, with an alternate allele frequency of < 4% in Indian population.

**Table 3:** Twelve SNPs with median *FLCN* expression values in Lung and Skin (exposed) from GTex, and their alternate allele frequencies

Sl. No. SNPs	COORD	SNP ID	Annotation	Ref allele	Alt allele frequency (South Asian/Asian)	Lung (median values of gene expression)				Skin-exposed (median values of gene expression)			
						REF	HET	ALT	*p*-value	REF	HET	ALT	*p*-value
SNP 1	17,236,986	rs41345949	5′ UTR	C	T = 0.03/0.05	− 0.06 (440)	0.27 (69)	N.A	1.10E−07	–	–	–	–
SNP 2	17,212,319	rs7218992	3′ UTR	C	A = –/0.06	0.126 (384)	− 0.37 (119)	− 0.91 (12)	8.90E−14	0.05 (456)	− 0.22 (137)	− 0.500 (12)	4.30E−07
SNP 3	17,213,262	rs12602675	3′ UTR	C	T = 0.08/0.08	0.014 (478)	− 0.29 (37)	N.A	0.00011	0.02 (564)	− 0.52 (40)	N.A	1.10E−09
SNP 4	17,221,501	rs3744124	Intronic	C	T = 0.11/0.12	0.07 (452)	− 0.44 (62)	N.A	2.40E−09	0.06 (529)	− 0.44 (76)	N.A	3.40E−18
SNP 5	17,226,931	rs6502565	Intronic	C	T = 0.12/0.13	0.068 (451)	− 0.44 (62)	N.A	1.80E−09	0.06 (528)	− 0.46 (76)	N.A	1.30E−17
SNP 6	17,226,956	rs76319098	Intronic	C	T = 0.12/0.13	0.05 (466)	− 0.47 (47)	N.A	1.80E−09	0.05 (548)	− 0.55 (56)	N.A	1.20E−17
SNP 7	17,228,852	rs79717038	Intronic	G	A = 0.12/0.13	0.06 (464)	− 0.47 (49)	N.A	2.70E−08	0.06 (543)	− 0.58 (61)	N.A	3.10E−19
SNP 8	17,214,314	rs8067893	Intronic	G	A = –/0.11	0.134 (340)	− 0.255 (151)	− 0.52 (24)	2.80E−12	0.08 (407)	− 0.23 (168)	0.03 (30)	1.70E−07
SNP 9	17,220,853	rs41323249	Intronic	C	T = 0.09/0.11	0.14 (359)	− 0.34 (139)	− 0.50 (17)	2.60E−14	0.07 (430)	− 0.12 (153)	− 0.73 (22)	4.40E− 08

COORD: Chromosomal co-ordinates, In expression columns (REF: Homozygous reference genotype, HET: Heterozygote genotype, ALT: Alternate allele genotype), REF allele: Reference allele, ALT allele: Alternate allele frequency in Indian (GenomeAsia100K) and South Asian (gnomAD) population, N.A.: Gene expression value not available due to none/very low sample size with particular genotype. The numbers in brackets indicate the number of samples harbouring that genotype

### PDT for family based test of association between *FLCN* mutations and BHDS

Although *c.1285delC* mutation is mostly observed in BHDS patients, but other less frequent mutations are also detected in different populations. Considering availability of family data, it is always better to do family based association study rather than case–control study, since family based study is more powerful. To consider less frequent *FLCN* mutations in patient population, we have performed family based association study including all pathogenic mutations. Five exonic pathogenic *FLCN* mutations (*c.634C* > *T*, *c.1150_1160del11*, *c.1285delC*, *c.1329_1332dupAGCC*, *c.1300* + *1G* > *A*) were tested for family-based association studies. Families F3 and F14 (with *c.1301-1G* > *A* and *c.1285delC* mutations, respectively) were not taken as they lacked the required conditions for the test. Calculating *Di* for the 8 families (Additional file 1: Table S10a), the test statistic (*T*) was found to be 1.83 (≥ 1.64 for significant association). Therefore, the pathogenic *FLCN* variants are significantly associated with BHDS.

#### Regulatory SNPs

Apart from regulatory SNP *rs41345949* observed in eQTL analysis, we also observed another *FLCN* SNP, *rs1708629*, affecting *FLCN* expression and disease penetrance [46]. TDT was also performed for these two SNPs for all families (Additional file 1: Tables S10b and S10c). Calculating for *Di*, where *i* = 1 to 6 (6 families) and *i* = 1 to 5 (5 families) for *rs1708629* and *rs41345949*, respectively; the test statistic (*T*) was found 0.93 for *rs1708629* and 0.85 for *rs41345949*. Therefore, these SNPs were not significantly associated with the BHDS.

### Mutational effects on FLCN: in silico study

**Fig. 2:**
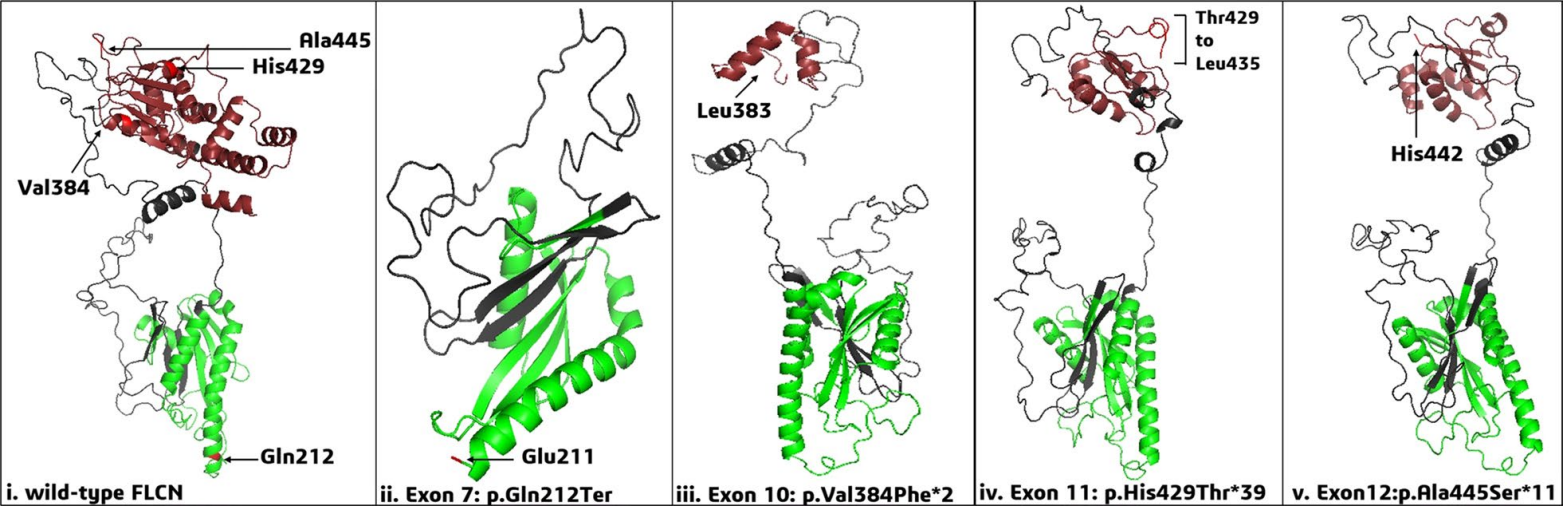
FLCN exonic mutations and their effect on protein structure and interacting proteins. **Green:** N-terminal/Longin of FLCN, **Brown:** C-terminal of FLCN. Structures of homology modelled monomers of FLCN protein—(i) wild-type FLCN with amino-acid residues which were affected by mutations; (ii) stop-gain mutant FLCN affecting chain termination after Glu211 (i.e., p.Gln212*); (iii) indel (11-nucleotide) mutant FLCN affecting chain termination after Leu383 (i.e., p.Val384Phe*2); (iv) indel (single-nucleotide) mutant FLCN, altering the frame from His429 to Thr429, and subsequently creating a stop-codon after Leu435 in the model (i.e., p.His429Thr*39); (v) four-nucleotide duplicate mutant FLCN, altering the frame and terminating the chain after His442 in the model (i.e., p.Ala445Ser*11)

FLCN protein has two distinct terminals: *Longin/N-terminal* (*Lys105*—*Cys265*) and C- terminal (*Pro344—Met566*) (Additional file 3: Fig. S5a). Three of four frame-shift (*fs*), protein-truncating mutations mapped to the C-terminal: p.Val384Phe*2, p.His429Thr*39, p.Ala445Ser*11, while, *p.Gln212Ter* mapped to N-terminal (Additional file 3: Fig. S5b) region. All four exonic mutations have pathogenic CADD scores (> 15) and resulted in a premature truncation of the protein (Fig. 2 and Additional file 1: Table S11). MaxEnt scores were 8.18 and 8.75 for the splice donor (*c.1300* + *1G* > *A*) and splice acceptor (*c.1301-1G* > *A*) mutations, and both had pathogenic CADD scores (34 and 33, respectively).

### Homology modelling of wild-type/mutant FLCN and effect on protein–protein interaction

Homology modeled complex of wild type (wt) or four mutant FLCN (p.Q212*, p.V384F*2, p.H429T*39, p.A445S*11), with wt-FNIP2, wt-RRAGA, and wt-RRAGC were validated by PROCHECK, with more than 98% residues in the allowed region of the Ramachandran plot (Additional file 1: Table S12), thus these monomers were accepted for further analyses. Macromolecular docking revealed HADDOCK scores of − 342.2 ± 0.0 and − 53.1 ± 10.3 for wt-FLCN-FNIP2-RRAGA-RRAGC and wt-FLCN-FNIP2 respectively. These data suggested the native FLCN-FNIP2 complex has weaker stability than the wild type four proteins complex. Each of four *FLCN* mutant monomers was docked with either wt-FNIP2-RRAGA-RRAGC or wt-FNIP2 and it resulted in lesser negative HADDOCK scores and low buried surface areas (BSA) than their native complexes (Additional file 1: Tables S13a and S13b). Less negative HADDOCK scores indicate a lower affinity between interacting partners, and low BSA indicates weaker protein stability. The HADDOCK score of exon-7 mutant-FLCN (p.Q212*) docked with wt-FNIP2-RRAGA-RRAGC had the least negative score (203.2 ± 30.5) than the other three mutant FLCN complexes (− 252.1 ± 20.6, − 264.6 ± 18.1, − 242.8 ± 2.8). On the other hand, in two protein docking complex, exon-10 mutant-*FLCN* (p.V384F*2) had least negative HADDOCK score (− 32.0 ± 19.4) followed by exon-11, exon-12 and exon-7 mutant FLCN (− 37.6 ± 11.5, −40.2 ± 7.4, and 47.9 ± 11.9, respectively). So, there was a considerable loss of interaction in all mutant complexes (Additional file 3: Figs. S6a and S6b).

### Copy number evaluation of *FLCN* by Taqman assays

**Fig. 3:**
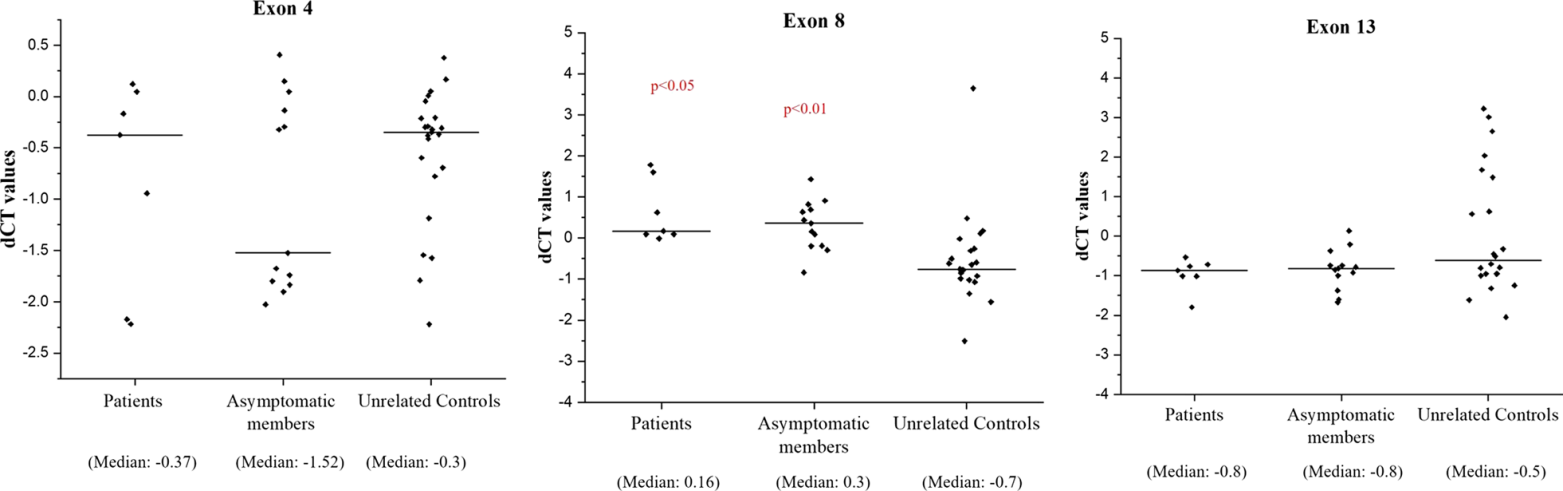
Normalized ∆Ct (dCT) values of patients, asymptomatic and unrelated healthy controls obtained from *FLCN* Taqman copy number assays for exons 4, 8 and 13. For calculation of *p*-values, 2^−∆ct^ values from patients and asymptomatic members were compared with unrelated controls. Patients and unrelated controls (*p*-value: 0.019), and asymptomatic members and unrelated controls (*p*-value: 0.008) had a significant difference in copy number only for exon 8. Non-significant *p*-values have not been shown in the figures

NGS data of *FLCN* revealed a difference in log transformed normalized read counts between patients and asymptomatic members particularly in four families (F3, F4, F9 and F10) (Additional file 3: Fig. S7). To validate these differences in normalized read counts, Taqman copy number assay was performed. Ct values were obtained from Taqman assays for exons 4, 8 and 13 of *FLCN*, for all members of the four families and 23 unrelated healthy controls. Normalized Ct values for the exons (dCT or ∆Ct) were obtained and transformed to 2^−∆ct^ for further analysis (Fig. 3). Analysis revealed a significant copy number difference for exon 8 in both patients and asymptomatic members compared to unrelated controls (*p*-values 0.019 and 0.008, respectively), but not between patients and asymptomatic members in any of the three exon assays (exon 4, 8 and 13). But non-parametric test for exon 4 assay and parametric unpaired t-test for exon 13 assay, in patients and asymptomatic members in comparison to unrelated controls did not result any significant copy number difference (Additional file 1: Table S14).

For better understanding of sample sizes used in different experiments and results; a summary flow chart (Additional file 4: Summary of the study) is added.

## Discussion

In this study, BHDS lung phenotype (PSP and/or multiple bilateral lung cysts) was found to be most prevalent followed by skin fibrofolliculomas and renal cysts/carcinoma (chromophobe) (Table 1). This observation is in accordance with several East Asian studies, where the lung phenotype is more common (87.3%), than skin lesions (36.7%) and kidney cancer (7.2%), unlike studies from Western countries [25]. These population-specific differences may be due to different genetic and/or environmental factors contributing to disease pathogenesis.

Twenty-seven of 31 patients were diagnosed with PSP, with recurrent pneumothoraces in 9 patients. Age of onset of pneumothorax recurrence in patients ranged from 15 to 59 years. We calculated the probability of recurrence of PSP in 27 patients based on a generalized estimate (GEE) in SPSS. The recurrence of PSP was taken as a dependant variable for ‘age of onset of first spontaneous pneumothorax’, while patient gender, presence/absence of family history, tobacco habits and presence of *FLCN* pathogenic mutations were considered as co-factors (Additional file 1: Table S15). Analysis revealed a significant association (p-value, 0.047) between the age of onset and PSP recurrence. The mean age of patients with recurrent PSP and single PSP in our cohort are 36 ± 13.01 and 40 ± 11.6 years respectively, with age of onset ≤ 25 years in two recurrent PSP patients. A recent study reported that patients with single PSP are significantly older (mean age: 38.9 ± 16) than patients with recurrent PSP (mean age: 29.7 ± 11) [47]. Therefore, age of onset is an important factor for PSP recurrences in patients.

Genotype-specific phenotypes were not observed in this study. Nineteen BHDS patients with pathogenic *FLCN* mutations (Table 2), showed lung phenotype with skin fibrofolliculomas in 4 patients and RCC/renal cysts in 2 patients, respectively (Fig. 1). One patient with *c.1285delC* mutation also presented breast fibroadenoma, which has been reported in another BHDS study with patients negative for pathogenic *FLCN* mutations [48]. Eleven of 19 patients (57.8%) from 5 families harboured known hotspot mutation—*c.1285delC*, which was also reported in most of the BHDS patients in other studies. Family based association (using PDT) between BHDS and *FLCN* mutations has been sparsely done in BHDS studies. Here, we observed that other rare mutation (i.e. novel, hotspot and splice donor mutations) (Table 2), were also significantly associated (Additional file 1: Table S10a) with BHDS in family based study.

Sixteen of 74 asymptomatic members also harbour pathogenic *FLCN* mutations. Mean age, at onset of BHDS phenotype, of 19 patients with pathogenic *FLCN* mutations was 44.1 ± 10.9 yrs, which is much higher than the mean age of 16 asymptomatic members (29.5 ± 20.7 yrs) with *FLCN* mutations. It suggests that, perhaps, a few of the asymptomatic members may manifest BHDS after few years. It may be noted, lower mean age of 16 asymptomatic members may be attributed to the presence of 7 minors (aged ≤ 15 years) in the asymptomatic group. All asymptomatic members also need to be clinically evaluated, since we observed an asymptomatic sibling with *c.1285del11* mutation harboring several small basal and bilateral lung cysts after clinical re-evaluation in a previous study [27]. Asymptomatic members with pathogenic mutations may harbour un-ruptured pulmonary cysts and abnormal epithelial/mesenchymal interactions in pleura [49] that may result in PSP later, when combined with other factors.

Homology modelling of interacting proteins with mutant FLCN containing novel and hotspot mutant *FLCN* significantly affected the protein structures (Fig. 2). Three protein-truncating pathogenic mutations (*p.Val384Phefs, p.His429Thrfs and p.Ala445Serfs*) were present in C-terminal of FLCN which interacts with FNIP1/2. The stop-gain mutation (*p.Gln212Ter*) was found in *longin* domain and it is crucial for Rag-mediated mTORC1 lysosomal activation. Their protein-interacting docking scores also indicated that the FLCN C-terminal mutations substantially reduced the protein stability in the FLCN-FNIP2 complex, while the stop-gain mutation (in *longin* domain) did so in the 4-protein complex (FLCN-FNIP2-RRAGA-RRAGC).

Large intragenic indels have been reported in BHDS patients [10, 50], however we could not study them using MLPA techniques to detect large intragenic deletion. We addressed the same, using our NGS data and Taqman copy number assay, to check any large *FLCN* deletion in samples. *FLCN* targeting NGS data showed a read count difference between patients and asymptomatic members of 4 families (F3, F4, F9 and F10). However, Taqman copy number assays for exons 4, 8 and 13 in *FLCN* could not detect any significant difference in copy numbers between patients and asymptomatic members. But a significant copy number difference was only observed when Taqman data for exon 8 in patients and asymptomatics were compared with those of unrelated controls (Fig. 3). But we could not validate this difference from NGS data, since we had not taken unrelated controls in the NGS study. Patients from five families (F6, F7, F8, F9 and F10) did not harbour any pathogenic *FLCN* mutation. This suggests that large deletion mutations at *FLCN* or mutations in other unknown genes may be associated with disease phenotype. Whole-genome sequencing (WGS) of patients may throw light in these mutations. Our results suggest for copy number differences at exon 8 when compared between BHDS patients and unrelated controls but not at exons 4 and 13, so, more BHDS families may be needed for the study to get real picture of copy number differences. Read count results from targeted amplicon NGS send a cautionary note, as their implied read-count differences between patients and asymptomatic members were not detected in subsequent Taqman assay. Therefore, another validation method is necessary for detection of copy number changes.

PSP or BHDS was not detected in the HPO analysis in patients from family F9 (Additional file 3: Fig. S2), although, the patients were clinically diagnosed as BHDS and have a positive family history of pulmonary cysts. Similarly, two patients from two different families were initially suspected to have LAMS, but genetic evaluation confirmed those as BHDS patients. Therefore, genetic evaluation is necessary for clinically diagnosed BHDS patients.

### FLCN Mutations and possible mode of molecular pathogenesis

Four protein truncating mutations (Table 2) were detected in this study and it is reported that misfolded *FLCN* proteins, due to truncating mutations, may lead to proteosomal degradation. The hotspot mutation, *c.1285dupC (H429Pfs)*, and several C-terminal missense mutations also destabilize the *FLCN*-*FNIP1*/2 binding [51]. BHDS patients in our cohort harbour lung cysts, and it was reported that loss of *FLCN* in murine alveolar cells resulted in dysfunctional activation of AMPK, leading to damaged lung function and apoptotic alveolar cell collapse [15].

Molecular docking analysis revealed that novel stop-gain mutation, p*.Gln212Ter* affects the FLCN-FNIP2-RRAGA-RRAGC binding and stability. It maps to the *longin* domain, where another residue, *p.Arg164*, is reported to be an important catalytic residue for GAP activity in mTORC1 activation in lysosomes [17]. Therefore, functional validation to know the role of the stop-gain mutation in this pathway is required. Three protein-truncating mutations were found in the C terminal region of FLCN, which also directly interacts with Rab7A, involved in lysosomal degradation of epidermal growth factor (EGFR). The study in a BHD-RCC cell line resulted in increased cell proliferation, migration and angiogenesis [20]. Therefore, the C terminal region of FLCN may have important, yet undiscovered, functions that may involve membrane trafficking in BHDS pulmonary phenotype.

## Conclusion

This is a first comprehensive genetic study from India with 15 BHDS families (31 patients and 74 asymptomatic individuals). We found 10 of 15 families (66.6%) harbour six pathogenic, protein-truncating *FLCN* mutations. Among these 6 mutations: two are novel, two were reported Clinvar pathogenic, one was hotspot and the remaining one was reported splice donor mutation. These mutations were significantly associated with disease phenotype in family based PDT study and found in key functional domains that might greatly affect protein binding and downstream signalling pathways. However, we did not find any pathogenic mutations at exons of *FLCN* in 5 clinically diagnosed BHDS families (F6, F7, F8, F9 and F10). Therefore, we suggest for whole genome sequencing of these patients to detect mutations in exons as well as introns at *FLCN* and/or other, yet, undescribed disease genes. Our findings suggest for presence of larger mutational spectrum in Indian patients.

## Supplementary Information

**Additional file 1**: Supplementary tables

**Additional file 2**: Supplementary methods

**Additional file 3**: Supplementary figures

**Additional file 4**: Summary of the study

## Data Availability

Additional data are provided in Supplementary materials.

## Abbreviations

BHDS: Birt–Hogg–Dubé syndrome
PSP: Primary spontaneous pneumothorax
RCC: Renal cell carcinoma
*FLCN*: *Folliculin*
NGS: Next-generation sequencing
FNIP: Folliculin-interacting protein
HPO: Human phenotype ontology
e-QTL: Expression quantitative trait loci
PDT: Pedigree disequilibrium test
RRAG: Ras-related GTP-binding protein
