# Supplementary material for: Genetic insight into Birt–Hogg–Dubé syndrome in Indian patients reveals novel mutations at FLCN
Source: Orphanet J Rare Dis. 2022 Apr 27;17:176. doi: 10.1186/s13023-022-02326-5 (PMC9044636; doi:10.1186/s13023-022-02326-5)
Supplement: Supplementary file 1 — Additional file 1: Supplementary tables [file 13023_2022_2326_MOESM1_ESM.docx]

**Additional file 1: Supplementary Tables**

**Table S1: Clinically diagnosed BHDS patients and related asymptomatic members enrolled from 15 families (n=105, patients and asymptomatic members)**

| **Family ID** | **Patient IDs with PSP or skin fibrofolliculomas** | **Asymptomatic member ID’s** | **Total no. of members** |
| --- | --- | --- | --- |
| F1 | F1-1, F1-2 | F1-3 to F1-8 | 8 |
| F2 | F2-9 | F2-10 to F2-12 | 4 |
| F3 | F3-13*, F3-14* | F3-15 to F3-17 | 5 |
| F4 | F4-18 | F4-19 to F4-22 | 5 |
| F5 | F5-25, F5-26, F5-28 | F5-23, F5-24, F5-27, F5-29 to F5-34 | 12 |
| F6 | F6-35* | F6-36 to F6-43 | 9 |
| F7 | F7-44*^#^, F7-45*, F7-46*, F7-47*, F7-48*, F7-49* | F7-50 to F7-55 | 12 |
| F8 | F8-56* | F8-57 to F8-59 | 4 |
| F9 | F9-60, F9-61, F9-62 | F9-63 to F9-66 | 7 |
| F10 | F10-67 | F10-68 to F10-69 | 3 |
| F11 | F11-70 | F11-71 to F11-76 | 7 |
| F12 | F12-77, F12-78* | F12-79 to F12-80 | 4 |
| F13 | F13-82^#^, F13-83, F13-84, F13-85 | F13-86 to F13-94 | 13 |
| F14 | F14-95*^#^ | F14-96 to F14-98 | 4 |
| F15 | F15-99, F15-101 | F15-100, F15-102 to F15-106 | 8 |
| **Total** | **31** | **74** | **105** |

**Note:** All patients except F7-49 had BHDS lung phenotype (PSP or lung cysts). Individual ID is given by Family number followed by their serial number as assigned during their enrolment in the study (e.g. F1-1 is index patient from family F1). Patient IDs have been anonymised by the research group. *Patients have skin fibrofolliculomas. #patients have renal cysts/carcinoma.

**Table S2: Patients (n=20) and asymptomatic members (n=15) from 11 families taken for targeted amplicon NGS of *FLCN***

| **Family ID** | **Patient ID** | **Asymptomatic member (AM) ID** | **Total** |
| --- | --- | --- | --- |
| F1* | F1-1* | - | 1 |
| F2 | F2-9 | F2-10, F2-12 | 3 |
| F3 | F3-13, F3-14 | F3-15 | 3 |
| F4 | F4-18 | F4-19 | 2 |
| F5 | F5-25, F5-26, F5-28 | F5-23, F5-24 | 5 |
| F6 | F6-35 | F6-36 to F6-40 | 6 |
| F7 | F7-44 to F7-48 | 0 | 5 |
| F8 | F8-56 | F8-57, F8-58 | 3 |
| F9 | F9-60, F9-61, F9-62 | F9-63 | 4 |
| F10 | F10-67 | F10-68 | 2 |
| F11** | F11-70** | - | 1 |
| **Total** | **20** | **15** | **35** |

*Result of *FLCN* mutation has been published in an earlier study (Ray *et al*., *Lung, 2015*), therefore mutation data from F1-1 taken as positive control. Other family members from family F1 were not included in this sequencing method.

** Patient F11-70 is the only individual included from family F11 due to logistic constraints

**Table S3a: Genomic coordinates and primer sequences for *FLCN* long PCR encompassing exons and UTRs, used in targeted amplicon NGS**

| Amplicon name | Genomic Coordinates | Sequences (5’ to 3’) | Amplicon Length (bp) | UTR’s and exons included |
| --- | --- | --- | --- | --- |
| FLN – A1 | chr17:17,237,734 to 17,231,271 | *CGAGTTCTGCAACCAACCTC* | 6464 | 3’UTR and exons 1, 2, 3 |
|  |  | *GTCCTGAGTGTTTGCTAGGC* |  |  |
| FLN – A2 | chr17:17,232,245 to 17,225,868 | *GCAAGGGGTACTCTGAGCAG* | 6378 | Exons 3, 4, 5 |
|  |  | *GAGACCCTGACACAAAAGAAGG* |  |  |
| FLN – A3 | chr17:17,223,045 to 17,217,787 | *CCTTGTTTGTCTCAGCTCATTG* | 5259 | Exons 7, 8, 9 |
|  |  | *GACACCTTAGGAGAGTCCAGC* |  |  |
| FLN – A4 | chr17:17,217,807 to 17,211,688 | *GCTGGACTCTCCTAAGGTGTC* | 6120 | Exons 10, 11, 12, 13, 14, and 5’UTR |
|  |  | *GCATAGGTACTCAACAGATGTCC* |  |  |

**Table S3b: Primers to amplify 14 exons and their flanking intronic regions (50bp) of *FLCN* for Sanger Sequencing method**

| PRIMERS | SEQUENCES |
| --- | --- |
| Exon 1 (UTR) | *5’- GTGTTGGGTGGTGGTACG-3’* |
|  | *5’- CCAACGAAAACTCGGACA -3’* |
| Exon 2 (UTR) | *5’- CTAAGCCATTCAGAACCCTG -3’* |
|  | *5’- GGCATTAAACTGCGAAAAGG -3’* |
| Exon 3 (UTR) | *5’- TCCCTCTCTGACTCCCACAC -3’* |
|  | *5’- GCCAAAGCCGCTAACTCTAG -3’* |
| Exon 4 | *5’- TCTCCTGGGCAGGAAGTC -3’* |
|  | *5’- GTCAGGATGAGCGGAAAGAA -3’* |
| Exon 5 | *5’- GCCCTGCTTCCCAACTAA -3’* |
|  | *5’- GCCCTGAGAGAGGACCAGT -3’* |
| Exon 6 | *5’- TTGTGCCAGCTGACTCTG -3’* |
|  | *5’- CCAGCTCTGAAGCCAAGA -3’* |
| Exon 7 | *5’- GTGGGACTGATCCTCCAG -3’* |
|  | *5’- CAGAGGCAGCAAGCAAAC -3’* |
| Exon 8 | *5’- GTGAGCGTCAGGTTTGCTTT -3’* |
|  | *5’- CTGCCAGGAGAGCAGACAG -3’* |
| Exon 9 | *5’- GGGCTGAAGTCACAGGAT -3’* |
|  | *5’- CCATGGGATGCCAACTAT -3’* |
| Exon 10 | *5’- GTCACGCTGAAAGCACTG -3’* |
|  | *5’- ACATCATCAGACCAGACCC -3’* |
| Exon 11 | *5’- CACTGTGGGCTGAGAGTCTG -3’* |
|  | *5’- CCTCTCCACAACCCATGA -3’* |
| Exon 12-13 | *5’- CCACTGACCTGGGATGAG -3’* |
|  | *5’- GGCCCAGCTCCTCTTTTG -3’* |
| Exon 14 | *5’- GCTCGAGGGATTGTGCTG -3’* |
|  | *5’- GCCCAGAAACTCTTGCTG -3’* |
| Exon 14 (UTR) | *5’- TCCTTGGAGAGACGACTAGG -3’* |
|  | *5’- CCGGCTGACTCACTGGTAT -3’* |

**Table S4: Patients and asymptomatic members in 4 families taken for Taqman *FLCN* copy number assays**

| **Family ID** | **Patient ID** | **Asymptomatic member (AM) ID** | **Total individuals** |
| --- | --- | --- | --- |
| F3 | F3-13, F3-14 | F3-15 to F3-17 | 5 |
| F4 | F4-18 | F4-19 to F4-22 | 5 |
| F9 | F9-60, F9-61, F9-62 | F9-63 to 66 | 7 |
| F10 | F10-67 | F10-68 & F10-69 | 3 |
| **N=4** | **N=7** | **N=13** | **N=20** |

**Table S5: Thirty one patients from 15 families with major or minor diagnostic clinical criteria for BHDS**

| **S. No** | **Family ID** | **Sample ID** | **Sex** | **Age Range (yr)** | ***Major Criteria*** | ***Minor Criteria*** | | |
| --- | --- | --- | --- | --- | --- | --- | --- | --- |
|  |  |  |  |  | **Fibrofolli-culomas** | **Pneumo-thorax or lung cysts** | **Renal cell carcinoma/renal cysts** | **First degree relative** |
| 1 | F1 | F1- 1 | F | 31-35 | absent | present | absent | present |
| 2 |  | F1- 2 | F | 31-35 | absent | present | absent | present |
| 3 | F2 | F2-9* | M | 46-50 | absent | present | absent | absent |
| 4 | F3^#^ | F3-13 | M | 41-45 | present | present | absent | present |
| 5 |  | F3-14 | M | 41-45 | present | present | absent | present |
| 6 | F4 | F4-18* | M | 41-45 | absent | present | absent | absent |
| 7 | F5 | F5-25 | F | 36-40 | absent | present | absent | present |
| 8 |  | F5-26 | M | 36-40 | absent | present | absent | present |
| 9 |  | F5-28 | M | 31-35 | absent | present | absent | present |
| 10 | F6^#^ | F6-35 | M | 61-65 | present | present | absent | absent |
| 11 | F7^#^ | F7-44^@^ | M | 56-60 | present | present | present | present |
| 12 |  | F7-45 | F | 61-65 | present | present | absent | present |
| 13 |  | F7-46 | M | 61-65 | absent | present | absent | present |
| 14 |  | F7-47 | F | 51-55 | present | present | absent | present |
| 15 |  | F7-48 | F | 75-80 | present | present | absent | present |
| 16 |  | F7-49 | M | 85-90 | present | unknown | absent | present |
| 17 | F8^#^ | F8-56 | F | 15-20 | present | present | absent | absent |
| 18 | F9 | F9-60 | M | 36-40 | absent | present | absent | present |
| 19 |  | F9-61 | F | 61-65 | absent | present | absent | present |
| 20 |  | F9-62 | M | 36-40 | absent | present | absent | present |
| 21 | F10 | F10-67* | F | 56-60 | absent | present | absent | absent |
| 22 | F11 | F11-70* | F | 41-45 | absent | present | absent | absent |
| 23 | F12^#^ | F12-77 | F | 71-75 | present | present | absent | present |
| 24 |  | F12-78 | M | 41-45 | absent | present | absent | present |
| 25 | F13 | F13-82^@^ | M | 56-60 | absent | present | present | present |
| 26 |  | F13-83 | M | 51-55 | absent | present | absent | present |
| 27 |  | F13-84 | M | 46-50 | absent | present | absent | present |
| 28 |  | F13-85 | M | 36-40 | absent | present | absent | present |
| 29 | F14^#^ | F14-95^@^ | F | 31-35 | present | present | present | Absent |
| 30 | F15 | F15-99 | M | 61-65 | absent | present | absent | present |
| 31 |  | F15-101 | F | 31-35 | absent | present | absent | present |

M: Male, F: Female. * Patients fulfilling only one criterion (BHDS-specific lung cysts or repeated pneumothoraces) but patient F11-70 has second degree relatives with BHDS.

^#^ Eleven patients (F3-13, F3-14, F6-35, F7-44, F7-45, F7-47, F7-48, F7-49, F8-56, F12-77, F14-95) have skin fibrofolliculomas,

^@^ Three patients (F7-44, F13-82, F14-95) have renal cysts/carcinoma.

**Table S6: Phenotype Ontology Analysis using Phenomizer for 31 patients**

| **Family ID** | **Patient ID** | **p- value** | **Score** | **OMIM** | **Description** | **Gene** |
| --- | --- | --- | --- | --- | --- | --- |
| F1 | F1-1 | 0.0053 | 4.4332 | 173600 | Primary Spontaneous Pneumothorax | *FLCN* |
|  | F1-2 | 0.0053 | 3.7429 | 173600 | Primary Spontaneous Pneumothorax | *FLCN* |
| F2 | F2-9 | 0.0053 | 3.7429 | 173600 | Primary Spontaneous Pneumothorax | *FLCN* |
| F3 | F3-13 | 0.0027 | 5.3443 | 135150 | Birt-Hogg-Dubé syndrome | *FLCN* |
|  | F3-14 | 0.3057 | 4.2794 | 135150 | Birt-Hogg-Dubé syndrome | *FLCN* |
| F4 | F4-18 | 0.016 | 3.4511 | 173600 | Primary Spontaneous Pneumothorax | *FLCN* |
| F5 | F5-25 | 0.016 | 3.4511 | 173601 | Primary Spontaneous Pneumothorax | *FLCN* |
|  | F5-26 | 0.1646 | 4.7636 | 158310 | Hereditary Mucoepthelial Dysplasia | - |
|  | F5-28 | 0.1646 | 4.7636 | 158310 | Hereditary Mucoepthelial Dysplasia | - |
| F6 | F6-35 | 0.016 | 2.8072 | 173600 | Primary Spontaneous Pneumothorax | *FLCN* |
| F7 | F7-44 | 0.0027 | 5.5242 | 135150 | Birt-Hogg-Dubé syndrome | *FLCN* |
|  | F7-45 | 0.0027 | 5.3443 | 135150 | Birt-Hogg-Dubé syndrome | *FLCN* |
|  | F7-46 | 0.0053 | 3.7429 | 173600 | Primary Spontaneous Pneumothorax | *FLCN* |
|  | F7-47 | 0.0053 | 5.0544 | 135150 | Birt-Hogg-Dubé syndrome | *FLCN* |
|  | F7-48 | 0.0027 | 5.3443 | 135150 | Birt-Hogg-Dubé syndrome | *FLCN* |
|  | F7-49 | 0.2532 | 5.4567 | 135150 | Birt-Hogg-Dubé syndrome | *FLCN* |
| F8 | F8-56 | 0.0053 | 6.0976 | 135150 | Birt-Hogg-Dubé syndrome | *FLCN* |
| F9 | F9-60 | 0.1646 | 4.7636 | 158310 | Hereditary Mucoepthelial Dysplasia | - |
|  | F9-61 | 0.1646 | 4.7636 | 158310 | Hereditary Mucoepthelial Dysplasia | - |
|  | F9-62 | 0.0266 | 4.4518 | 610913 | ILD due to surfactant protein C deficiency | SFTPC |
| F10 | F10-67 | 0.016 | 2.8072 | 173600 | Primary Spontaneous Pneumothorax | *FLCN* |
| F11 | F11-70 | 0.0053 | 4.4332 | 173600 | Primary Spontaneous Pneumothorax | *FLCN* |
| F12 | F12-77 | 0.0053 | 4.652 | 173600 | Primary Spontaneous Pneumothorax | *FLCN* |
|  | F12-78 | 0.0053 | 3.7429 | 173600 | Primary Spontaneous Pneumothorax | *FLCN* |
| F13 | F13-82 | 0.0018 | 4.3871 | 135150 | Birt-Hogg-Dubé syndrome | *FLCN* |
|  | F13-83 | 0.0053 | 4.652 | 173600 | Primary Spontaneous Pneumothorax | *FLCN* |
|  | F13-84 | 0.0053 | 4.652 | 173600 | Primary Spontaneous Pneumothorax | *FLCN* |
|  | F13-85 | 0.0053 | 4.4332 | 173600 | Primary Spontaneous Pneumothorax | *FLCN* |
| F14 | F14-95 | 0.0053 | 5.5034 | 135150 | Birt-Hogg-Dubé syndrome | *FLCN* |
| F15 | F15-99 | 0.1646 | 4.7636 | 158310 | Hereditary Mucoepthelial Dysplasia | - |
|  | F15-101 | 0.0053 | 3.5465 | 173600 | Primary Spontaneous Pneumothorax | *FLCN* |

Note: ILD: interstitial lung disease. Patients of most of the families were diagnosed with BHDS or PSP, therefore, all patients were considered to have mutations at *FLCN* in this analysis. After sequencing it was found that patients in families F6, F7, F8, F9 and F10 did not have *FLCN* mutations.

**Table S7: Total Read Count for 20 patients and 15 asymptomatic members obtained from *FLCN* targeted amplicon NGS data (post quality filters)**

| **Sample ID** | **Patient/Asymptomatic member** | **Total Read Count (SeqMonk)** |
| --- | --- | --- |
| F1-1 | Patient | 7492195 |
| F2-9 | Patient | 7145316 |
| F2-10 | Asymptomatic member | 6742544 |
| F2-12 | Asymptomatic member | 5171128 |
| F3-13 | Patient | 5928218 |
| F3-14 | Patient | 5510277 |
| F3-15 | Asymptomatic member | 6196936 |
| F4-18 | Patient | 7614903 |
| F4-19 | Asymptomatic member | 8028362 |
| F5-23 | Asymptomatic member | 5132578 |
| F5-24 | Asymptomatic member | 7808531 |
| F5-25 | Patient | 8367280 |
| F5-26 | Patient | 7863282 |
| F5-28 | Patient | 8663063 |
| F6-35 | Patient | 7957945 |
| F6-36 | Asymptomatic member | 6794231 |
| F6-37 | Asymptomatic member | 6825705 |
| F6-38 | Asymptomatic member | 7624874 |
| F6-39 | Asymptomatic member | 5776490 |
| F6-40 | Asymptomatic member | 6612516 |
| F7-44 | Patient | 9033619 |
| F7-45 | Patient | 7346030 |
| F7-46 | Patient | 6655348 |
| F7-47 | Patient | 7955265 |
| F7-49 | Patient | 7379168 |
| F8-56 | Patient | 6311258 |
| F8-57 | Asymptomatic member | 9789782 |
| F8-58 | Asymptomatic member | 8156624 |
| F9-60 | Patient | 6556172 |
| F9-61 | Patient | 6174414 |
| F9-62 | Patient | 8032191 |
| F9-63 | Asymptomatic member | 6827487 |
| F10-67 | Patient | 6919623 |
| F10-68 | Asymptomatic member | 8557839 |
| F11-70 | Patient | 7266443 |

**Note:** The reads were aligned to human reference (hg38) and after various quality filters, an average of 7.2 million reads was obtained, while the average depth of coverage of all samples was ~18,000x.

**Table S8a: Non-pathogenic/non-coding SNV’s in *FLCN* found by NGS**(Presence of mutations in **brown in** 3’ UTR, **green in** intron and **blue in** 5’UTR)

| **S. No.** | **COORD** | **ID** | **HGVS** | **Annotation** | **REF** | **ALT** | **Benign/ published reports** | **F2** | **F3** | **F4** | **F5** | **F6** | **F7** | **F8** | **F9** | **F10** | **F1-1** | **F11-70** |
| --- | --- | --- | --- | --- | --- | --- | --- | --- | --- | --- | --- | --- | --- | --- | --- | --- | --- | --- |
| 1 | 17212251 | *rs1451192210* | *c.*1404C>T* | 3' UTR | *G* | *A* |  |  |  |  |  |  |  |  |  |  |  |  |
| 2 | 17212252 | *rs7218795* | *c.*1403T>C* | 3' UTR | *A* | *G* | benign |  |  |  |  |  |  |  |  |  |  |  |
| 3 | 17212319 | *rs7218992* | *c.*1336G>A* | 3' UTR | *C* | *A* | benign |  |  |  |  |  |  |  |  |  |  |  |
| 4 | 17213098 | *rs3803761* | *c.*557T>C* | 3' UTR | *A* | *G* | benign |  |  |  |  |  |  |  |  |  |  |  |
| 5 | 17213230 | *rs7224335* | *c.*425G>A* | 3' UTR | *C* | *T* | benign |  |  |  |  |  |  |  |  |  |  |  |
| 6 | 17213262 | *rs12602675* | *c.*393G>A* | 3' UTR | *C* | *T* | benign |  |  |  |  |  |  |  |  |  |  |  |
| 7 | 17213299 | *rs7224474* | *c.*356G>T* | 3' UTR | *C* | *A* | benign |  |  |  |  |  |  |  |  |  |  |  |
| 8 | 17214195 | *rs8068606* | *c.1539-339A>C* | intron variant | *T* | *G* |  |  |  |  |  |  |  |  |  |  |  |  |
| 9 | 17214314 | *rs8067893* | *c.1539-458C>T* | intron variant | *G* | *A* |  |  |  |  |  |  |  |  |  |  |  |  |
| 10 | 17214401 | *-* | *c.1539-545G>A* | intron variant | *C* | *T* |  |  |  |  |  |  |  |  |  |  |  |  |
| 11 | 17215128 | *rs34235236* | *c.1433-38A>G* | intron variant | *T* | *C* | benign |  |  |  |  |  |  |  |  |  |  |  |
| 12 | 17215375 | *rs34311146* | *c.1301-59C>T* | intron variant | *G* | *A* | published |  |  |  |  |  |  |  |  |  |  |  |
| 13 | 17215386 | *rs544930629* | *c.1301-70C>T* | intron variant | *G* | *A* |  |  |  |  |  |  |  |  |  |  |  |  |
| 14 | 17215879 | *rs572265105* | *c.1300+501A>G* | intron variant | *T* | *C* |  |  |  |  |  |  |  |  |  |  |  |  |
| 15 | 17216890 | *rs7208065* | *c.1176+179A>G* | intron variant | *T* | *C* | published |  |  |  |  |  |  |  |  |  |  |  |
| 16 | 17217030 | *rs41424546* | *c.1176+39G>A* | intron variant | *C* | *T* | published |  |  |  |  |  |  |  |  |  |  |  |
| 17 | 17217354 | *rs4985705* | *c.1063-172C>G* | intron variant | *G* | *C* | published |  |  |  |  |  |  |  |  |  |  |  |
| 18 | 17217498 | *rs4985752* | *c.1063-316A>G* | intron variant | *T* | *C* |  |  |  |  |  |  |  |  |  |  |  |  |
| 19 | 17218310 | *rs8080386* | *c.1062+709A>G* | intron variant | *T* | *C* |  |  |  |  |  |  |  |  |  |  |  |  |
| 20 | 17218972 | *rs8065572* | *c.1062+47G>A* | intron variant | *C* | *T* |  |  |  |  |  |  |  |  |  |  |  |  |
| 21 | 17219013 | *rs8065832* | *c.1062+6C>T* | intron variant | *G* | *A* | benign |  |  |  |  |  |  |  |  |  |  |  |
| 22 | 17219819 | *rs2018781* | *c.872-610C>G* | intron variant | *G* | *C* | published |  |  |  |  |  |  |  |  |  |  |  |
| 23 | 17220853 | *rs41323249* | *c.871+684G>A* | intron variant | *C* | *T* | published |  |  |  |  |  |  |  |  |  |  |  |
| 24 | 17221311 | *rs41400246* | *c.871+226G>A* | intron variant | *C* | *T* | published |  |  |  |  |  |  |  |  |  |  |  |
| 25 | 17221501 | *rs3744124* | *c.871+36G>A* | intron variant | *C* | *T* | benign |  |  |  |  |  |  |  |  |  |  |  |
| 26 | 17222119 | *rs1708622* | *c.779+382T>C* | intron variant | *A* | *G* |  |  |  |  |  |  |  |  |  |  |  |  |
| 27 | 17222281 | *rs1736221* | *c.779+220T>C* | intron variant | *A* | *G* |  |  |  |  |  |  |  |  |  |  |  |  |
| 28 | 17222727 | *rs2292527* | *c.619-66C>T* | intron variant | *G* | *A* |  |  |  |  |  |  |  |  |  |  |  |  |
| 29 | 17226117 | *rs41525346* | *c.396+59T>C* | intron variant | *A* | *G* | benign |  |  |  |  |  |  |  |  |  |  |  |
| 30 | 17226610 | *rs1708620* | *c.250-288G>A* | intron variant | *C* | *T* | published |  |  |  |  |  |  |  |  |  |  |  |
| 31 | 17226729 | *rs1708619* | *c.250-407G>A* | intron variant | *C* | *T* |  |  |  |  |  |  |  |  |  |  |  |  |
| 32 | 17226923 | *rs1736216* | *c.250-601C>T* | intron variant | *G* | *A* |  |  |  |  |  |  |  |  |  |  |  |  |
| 33 | 17226931 | *rs6502565* | *c.250-609G>A* | intron variant | *C* | *T* |  |  |  |  |  |  |  |  |  |  |  |  |
| 34 | 17226956 | *rs76319098* | *c.250-634G>A* | intron variant | *C* | *T* |  |  |  |  |  |  |  |  |  |  |  |  |
| 35 | 17227493 | *rs373903620* | *c.249+396G>A* | intron variant | *C* | *T* |  |  |  |  |  |  |  |  |  |  |  |  |
| 36 | 17227704 | *rs55836267* | *c.249+185A>C* | intron variant | *T* | *G* |  |  |  |  |  |  |  |  |  |  |  |  |
| 37 | 17228404 | *rs75336342* | *c.-24-243G>A* | intron variant | *C* | *T* |  |  |  |  |  |  |  |  |  |  |  |  |
| 38 | 17228555 | *rs1708618* | *c.-24-394A>G* | intron variant | *T* | *C* |  |  |  |  |  |  |  |  |  |  |  |  |
| 39 | 17228589 | *rs538804082* | *c.-24-428G>T* | intron variant | *C* | *A* |  |  |  |  |  |  |  |  |  |  |  |  |
| 40 | 17228852 | *rs79717038* | *c.-24-691C>T* | intron variant | *G* | *A* |  |  |  |  |  |  |  |  |  |  |  |  |
| 41 | 17228888 | *rs1736215* | *c.-24-727C>T* | intron variant | *G* | *A* | published |  |  |  |  |  |  |  |  |  |  |  |
| 42 | 17229019 | *rs1736214* | *c.-24-858G>C* | intron variant | *C* | *G* |  |  |  |  |  |  |  |  |  |  |  |  |
| 43 | 17229099 | *rs565915341* | *c.-24-938C>T* | intron variant | *G* | *A* |  |  |  |  |  |  |  |  |  |  |  |  |
| 44 | 17229304 | *rs1708617* | *c.-24-1143T>C* | intron variant | *A* | *G* |  |  |  |  |  |  |  |  |  |  |  |  |
| 45 | 17229807 | *rs1613416* | *c.-24-1646C>T* | intron variant | *G* | *A* |  |  |  |  |  |  |  |  |  |  |  |  |
| 46 | 17230965 | *rs12602831* | *c.-25+829C>T* | intron variant | *G* | *T* |  |  |  |  |  |  |  |  |  |  |  |  |
| 47 | 17231193 | *rs12602871* | *c.-25+601C>T* | intron variant | *G* | *A* |  |  |  |  |  |  |  |  |  |  |  |  |
| 48 | 17231214 | *rs1736213* | *c.-25+580A>C* | intron variant | *T* | *G* | published |  |  |  |  |  |  |  |  |  |  |  |
| 49 | 17231694 | *rs1736212* | *c.-25+100C>G* | intron variant | *G* | *C* | published |  |  |  |  |  |  |  |  |  |  |  |
| 50 | 17232108 | *rs1736211* | *c.-113-226T>C* | intron variant | *A* | *G* |  |  |  |  |  |  |  |  |  |  |  |  |
| 51 | 17233101 | *rs76045368* | *c.-227-200G>A* | intron variant | *C* | *T* |  |  |  |  |  |  |  |  |  |  |  |  |
| 52 | 17233301 | *rs2349865* | *c.-227-400T>A* | intron variant | *A* | *T* |  |  |  |  |  |  |  |  |  |  |  |  |
| 53 | 17233347 | *rs34518797* | *c.-227-446G>A* | intron variant | *C* | *T* |  |  |  |  |  |  |  |  |  |  |  |  |
| 54 | 17233379 | *rs11078378* | *c.-227-478C>G* | intron variant | *G* | *C* |  |  |  |  |  |  |  |  |  |  |  |  |
| 55 | 17233577 | *rs10459910* | *c.-227-676G>A* | intron variant | *C* | *T* |  |  |  |  |  |  |  |  |  |  |  |  |
| 56 | 17233992 | *rs8079562* | *c.-227-1091C>G* | intron variant | *G* | *A* |  |  |  |  |  |  |  |  |  |  |  |  |
| 57 | 17234361 | *rs375429199* | *c.-227-1460C>T* | intron variant | *G* | *A* |  |  |  |  |  |  |  |  |  |  |  |  |
| 58 | 17234497 | *rs8079971* | *c.-227-1596T>C* | intron variant | *A* | *G* |  |  |  |  |  |  |  |  |  |  |  |  |
| 59 | 17234845 | *rs8065774* | *c.-227-1944G>A* | intron variant | *C* | *T* |  |  |  |  |  |  |  |  |  |  |  |  |
| 60 | 17235067 | *rs8066090* | *c.-228+1845T>C* | intron variant | *A* | *G* |  |  |  |  |  |  |  |  |  |  |  |  |
| 61 | 17235918 | *rs41337846* | *c.-228+994A>G* | intron variant | *T* | *C* | published |  |  |  |  |  |  |  |  |  |  |  |
| 62 | 17236983 | *rs1708629* | *c.-299C>T* | 5' UTR | *G* | *A* | benign |  |  |  |  |  |  |  |  |  |  |  |
| 63 | 17236986 | *rs41345949* | *c.-302G>A* | 5' UTR | *C* | *T* | benign |  |  |  |  |  |  |  |  |  |  |  |
| 64 | 17237171 | *rs1736209* | *c.-287G>C* | 5' UTR | *C* | *G* | benign |  |  |  |  |  |  |  |  |  |  |  |

**Note:** COORD: chromosomal position. Coloured boxes indicate presence of the variant in that family. 3’ UTR variants are denoted by brown, 5’ UTR variants in blue, and intronic variants in green.

**Table S8b: Non-coding indels in *FLCN* found by NGS** (Presence of mutation in **green:** intron)

| **S. No.** | **COORD** | **ID** | **HGVS** | **Annotation** | **REF** | **ALT** | **Benign/ published reports** | **F2** | **F3** | **F4** | **F5** | **F6** | **F7** | **F8** | **F9** | **F 10** | **F1-1** | **F 11-70** |
| --- | --- | --- | --- | --- | --- | --- | --- | --- | --- | --- | --- | --- | --- | --- | --- | --- | --- | --- |
| 1 | 17214142 | *rs10681411* | *c.1539-290_ 1539-287dup* | intronic | *G* | *-/G* | - |  |  |  |  |  |  |  |  |  |  |  |
| 2 | 17214701 | *rs146159727* | *c.1538+281_ 1538+284del* | intronic | *CATT* | *CATT/-* | - |  |  |  |  |  |  |  |  |  |  |  |
| 3 | 17227281 | *rs113749420* | *c.249+604_ 249+607del* | intronic | *ACCC* | *ACCC/-* | - |  |  |  |  |  |  |  |  |  |  |  |
| 4 | 17228584 | *rs200365194* | *c.-24-425_ -24-424del* | intronic | *GG* | *GG/-* | - |  |  |  |  |  |  |  |  |  |  |  |
| 5 | 17228592 | *rs879808064* | *c.-24-435_ -24-432del* | intronic | *GG* | *GG/-* | - |  |  |  |  |  |  |  |  |  |  |  |
| 6 | 17229504 | *rs66460366* | *c.-24-1345_ -24-1344del* | intronic | *AT* | *AT/-* | - |  |  |  |  |  |  |  |  |  |  |  |

**Note:** COORD: chromosomal position. Coloured boxes indicate presence of the variant in that family. 3’ UTR variants are denoted by brown, 5’ UTR variants in blue, and intronic variants in green

**Table S9a: Normalized gene expression values (median) of *FLCN* associated with 45 different SNPs (found in our cohort) common with *FLCN* SNPs in e-QTL data of Gtex database in Lung and Skin (exposed and unexposed) tissues**

| **S. No.** | **COORD** | **ID** | **HGVS** | **REF** | **ALT - FREQ (dbSNP)** | **LUNG genotypes (normalized expression median values)** | | | |
| --- | --- | --- | --- | --- | --- | --- | --- | --- | --- |
|  |  |  |  |  |  | **p-value** | **REF** | **HET** | **ALT** |
| 1 | 17236983 | *rs1708629* | 5' UTR variant | G | A=0.41 | 1.90E-82 | -0.65(144) | -0.014(241) | 0.69(130) |
| 2 | 17236986 | *rs41345949* | 5' UTR variant | C | T=0.05 | 1.10E-07 | -0.06(440) | 0.27(69) | N.A |
| 3 | 17237171 | *rs1736209* | 5' UTR variant | C | G=0.72 | 7.30E-23 | -0.65(33) | -0.20(220) | 0.28(262) |
| 4 | 17212252 | *rs7218795* | 3' UTR variant | A | G=0.66 | 8.20E-25 | -0.67(40) | -0.22(213) | 0.34 (262) |
| 5 | 17212319 | *rs7218992* | 3' UTR variant | C | A=0.06 | 8.90E-14 | 0.126(384) | -0.37 (119) | -0.91 (12) |
| 6 | 17213098 | *rs3803761* | 3' UTR variant | A | G=0.65 | 3.00E-25 | -0.67(42) | -0.21(213) | 0.32 (260) |
| 7 | 17213262 | *rs12602675* | 3' UTR variant | C | T=0.08 | 0.00011 | 0.014(478) | -0.29(37) | N.A |
| 8 | 17216890 | *rs7208065* | intron variant | T | C=0.48 | 7.10E-86 | -0.782(120) | -0.02(260) | 0.724(135) |
| 9 | 17217030 | *rs41424546* | intron variant | C | T=0.108 | 6.90E-14 | 0.13(396) | -0.37(109) | -0.4(10) |
| 10 | 17217354 | *rs4985705* | intron variant | G | C=0.47 | 4.50E-88 | -0.79(121) | -0.014(257) | 0.71(137) |
| 11 | 17217498 | *rs4985752* | intron variant | T | C= 0.41 | 2.80E-81 | -0.66(144) | 0 (243) | 0.71(128) |
| 12 | 17218310 | *rs8080386* | intron variant | T | C=0.60 | 2.10E-55 | -0.79(83) | -0.08(266) | 0.5(166) |
| 13 | 17219013 | *rs8065832* | intron variant | G | A=0.43 | 9.30E-65 | -0.66(147) | 0.08 (259) | 0.65 (109) |
| 14 | 17219819 | *rs2018781* | intron variant | G | C=0.73 | 4.90E-21 | -0.65(25) | -0.26(211) | 0.27(279) |
| 15 | 17221501 | *rs3744124* | intron variant | C | T=0.12 | 2.40E-09 | 0.07 (452) | -0.44 (62) | N.A |
| 16 | 17222119 | *rs1708622* | intron variant | A | G=0.74 | 8.10E-20 | -0.65(22) | -0.26(209) | 0.25(284) |
| 17 | 17222281 | *rs1736221* | intron variant | A | G=0.8 | 2.10E-55 | -0.79(83) | -0.089(266) | 0.57(166) |
| 18 | 17226610 | *rs1708620* | intron variant | C | T=0.43 | 3.60E-66 | -0.65(146) | 0.08(258) | 0.65(111) |
| 19 | 17226729 | *rs1708619* | intron variant | C | T= 0.44 | 9.90E-66 | -0.65(145) | 0.08(259) | 0.65(111) |
| 20 | 17226923 | *rs1736216* | intron variant | G | A=0.70 | 6.50E-11 | -0.46(40) | -0.12(224) | 0.16(251) |
| 21 | 17226931 | *rs6502565* | intron variant | C | T=0.13 | 1.80E-09 | 0.068(451) | -0.44(62) | N.A |
| 22 | 17226956 | *rs76319098* | intron variant | C | T=0.13 | 1.80E-09 | 0.05(466) | -0.47(47) | N.A |
| 23 | 17228555 | *rs1708618* | intron variant | T | C=0.47 | 1.10E-96 | -0.78(126) | 0.009(253) | 0.65(136) |
| 24 | 17228852 | *rs79717038* | intron variant | G | A=0.13 | 2.70E-08 | 0.06 (464) | -0.47(49) | N.A |
| 25 | 17228888 | *rs1736215* | intron variant | G | A=0.46 | 2.30E-90 | -0.76(119) | -0.02(256) | 0.69(140) |
| 26 | 17229019 | *rs1736214* | intron variant | C | G=0.46 | 2.10E-88 | -0.8(118) | -0.014(257) | 0.65(140) |
| 27 | 17229304 | *rs1708617* | intron variant | A | G=0.72 | 9.10E-23 | -0.65(34) | -0.20(218) | 0.29(263) |
| 28 | 17229807 | *rs1613416* | intron variant | G | A=0.47 | 2.30E-90 | -0.78(120) | -0.01(254) | 0.66(141) |
| 29 | 17231214 | *rs1736213* | intron variant | T | G=0.48 | 2.00E-90 | -0.79(119) | -0.01(256) | 0.69(140) |
| 30 | 17231694 | *rs1736212* | intron variant | G | C=0.72 | 9.10E-23 | -0.65(34) | -0.20(218) | 0.29(263) |
| 31 | 17232108 | *rs1736211* | intron variant | A | G=0.74 | 1.70E-20 | -0.65(24) | -0.24(203) | 0.23(288) |
| 32 | 17233301 | *rs2349865* | intron variant | A | T=0.25 | 8.80E-23 | -0.65(34) | -0.20(219) | 0.28 (262) |
| 33 | 17233379 | *rs11078378* | intron variant | G | C=0.74 | 3.90E-20 | -0.65(23) | -0.24(205) | 0.22(287) |
| 34 | 17214314 | *rs8067893* | intron variant | G | A=0.11 | 2.80E-12 | 0.134(340) | -0.255(151) | -0.52 (24) |
| 35 | 17220853 | *rs41323249* | intron variant | C | T=0.12 | 2.60E-14 | 0.14(359) | -0.34(139) | -0.50(17) |
| 36 | 17221311 | *rs41400246* | intron variant | C | T=0.10 | 2.10E-14 | 0.13(393) | -0.37(112) | -0.4 (10) |
| 37 | 17226117 | *rs41525346* | intron variant | A | G=0.08 | 9.90E-08 | 0.08(436) | -0.43(76) | N.A |
| 38 | 17227704 | *rs55836267* | intron variant | T | G= 0.12 | 2.60E-14 | 0.14(359) | -0.34(139) | -0.50(17) |
| 39 | 17230965 | *rs12602831* | intron variant | G | T=0.25 | 7.10E-24 | 0.20(356) | -0.34(140) | -0.42(19) |
| 40 | 17231193 | *rs12602871* | intron variant | G | A=0.25 | 7.10E-24 | 0.20(356) | -0.34(140) | -0.42(19) |
| 41 | 17233101 | *rs76045368* | intron variant | C | T=0.25 | 3.30E-25 | 0.24(331) | -0.35(160) | -0.46(24) |
| 42 | 17233347 | *rs34518797* | intron variant | C | T=0.25 | 3.30E-25 | 0.24(331) | -0.35(160) | -0.46(24) |
| 43 | 17233992 | *rs8079562* | intron variant | G | A=0.25 | 3.30E-25 | 0.24(331) | -0.35(160) | -0.46(24) |
| 44 | 17234845 | *rs8065774* | intron variant | C | T=0.24 | 3.30E-25 | 0.24(331) | -0.35(160) | -0.46(24) |
| 45 | 17235918 | *rs41337846* | intron variant | T | C=0.24 | 6.60E-25 | 0.21(354) | -0.35(142) | -0.42(19) |

| **S.No.** | **COORD** | **ID** | **HGVS** | **REF** | **ALT - FREQ (dbSNP)** | **SKIN (exposed) genotypes (normalized expression median values)** | | | |
| --- | --- | --- | --- | --- | --- | --- | --- | --- | --- |
|  |  |  |  |  |  | **p-value** | **REF** | **HET** | **ALT** |
| 1 | 17236983 | *rs1708629* | 5' UTR variant | G | A=0.41 | 1.80E-55 | -0.38(162) | 0.01(263) | 0.27(180) |
| 2 | 17236986 | *rs41345949* | 5' UTR variant | C | T=0.05 | - | - | - | - |
| 3 | 17237171 | *rs1736209* | 5' UTR variant | C | G=0.72 | 1.00E-11 | 0.06(33) | -0.17(233) | 0.14(339) |
| 4 | 17212252 | *rs7218795* | 3' UTR variant | A | G=0.66 | 1.70E-20 | -0.3(44) | -0.19(234) | 0.19(327) |
| 5 | 17212319 | *rs7218992* | 3' UTR variant | C | A=0.06 | 4.30E-07 | 0.05(456) | -0.22(137) | -0.500(12) |
| 6 | 17213098 | *rs3803761* | 3' UTR variant | A | G=0.65 | 7.30E-20 | -0.20(46) | -0.19(235) | 0.19(324) |
| 7 | 17213262 | *rs12602675* | 3' UTR variant | C | T=0.08 | 1.10E-09 | 0.02(564) | -0.52(40) | N.A |
| 8 | 17216890 | *rs7208065* | intron variant | T | C=0.48 | 1.70E-54 | -0.62(129) | 0.03(296) | 0.30(180) |
| 9 | 17217030 | *rs41424546* | intron variant | C | T=0.108 | 1.30E-10 | 0.08(466) | -0.29(126) | -1.11(13) |
| 10 | 17217354 | *rs4985705* | intron variant | G | C=0.47 | 1.40E-57 | -0.63(130) | 0.03(292) | 0.29(183) |
| 11 | 17217498 | *rs4985752* | intron variant | T | C= 0.41 | 4.10E-56 | -0.46(161) | 0.04(268) | 0.28(176) |
| 12 | 17218310 | *rs8080386* | intron variant | T | C=0.60 | 1.70E-30 | -0.55(85) | -0.09(302) | 0.28(218) |
| 13 | 17219013 | *rs8065832* | intron variant | G | A=0.43 | 4.30E-45 | -0.48(162) | 0.04(294) | 0.28(149) |
| 14 | 17219819 | *rs2018781* | intron variant | G | C=0.73 | 9.50E-14 | -0.06(26) | -0.23(226) | 0.17(353) |
| 15 | 17221501 | *rs3744124* | intron variant | C | T=0.12 | 3.40E-18 | 0.06(529) | -0.44(76) | N.A |
| 16 | 17222119 | *rs1708622* | intron variant | A | G=0.74 | 1.80E-13 | -0.09(23) | -0.21(222) | 0.155(360) |
| 17 | 17222281 | *rs1736221* | intron variant | A | G=0.8 | 1.70E-30 | -0.55(85) | -0.09(302) | 0.28(218) |
| 18 | 17226610 | *rs1708620* | intron variant | C | T=0.43 | 9.60E-45 | -0.47(161) | 0.03(292) | 0.28(152) |
| 19 | 17226729 | *rs1708619* | intron variant | C | T= 0.44 | 6.70E-45 | -0.47(160) | 0.03(292) | 0.28(153) |
| 20 | 17226923 | *rs1736216* | intron variant | G | A=0.70 | 6.60E-08 | -0.03(44) | -0.15(242) | 0.13(319) |
| 21 | 17226931 | *rs6502565* | intron variant | C | T=0.13 | 1.30E-17 | 0.06(528) | -0.46(76) | N.A |
| 22 | 17226956 | *rs76319098* | intron variant | C | T=0.13 | 1.20E-17 | 0.05(548) | -0.55(56) | N.A |
| 23 | 17228555 | *rs1708618* | intron variant | T | C=0.47 | 2.50E-63 | -0.65(135) | 0.05(291) | 0.31(179) |
| 24 | 17228852 | *rs79717038* | intron variant | G | A=0.13 | 3.10E-19 | 0.06(543) | -0.58(61) | N.A |
| 25 | 17228888 | *rs1736215* | intron variant | G | A=0.46 | 5.30E-57 | -0.63(128) | 0.01(291) | 0.28(186) |
| 26 | 17229019 | *rs1736214* | intron variant | C | G=0.46 | 4.00E-56 | -0.59(126) | 0.01(290) | 0.29(189) |
| 27 | 17229304 | *rs1708617* | intron variant | A | G=0.72 | 7.70E-12 | 0.08(32) | -0.19(233) | 0.14(340) |
| 28 | 17229807 | *rs1613416* | intron variant | G | A=0.47 | 1.30E-56 | -0.62(129) | 0.01(288) | 0.28(188) |
| 29 | 17231214 | *rs1736213* | intron variant | T | G=0.48 | 5.50E-58 | -0.63(128) | 0.016(289) | 0.28(188) |
| 30 | 17231694 | *rs1736212* | intron variant | G | C=0.72 | 7.70E-12 | 0.08(32) | -0.19(233) | 0.14(340) |
| 31 | 17232108 | *rs1736211* | intron variant | A | G=0.74 | 8.40E-13 | -0.04(25) | -0.19(214) | 0.14(366) |
| 32 | 17233301 | *rs2349865* | intron variant | A | T=0.25 | 5.10E-12 | 0.08(33) | -0.19(233) | 0.14 (339) |
| 33 | 17233379 | *rs11078378* | intron variant | G | C=0.74 | 8.40E-13 | -0.04(25) | -0.19(214) | 0.14(366) |
| 34 | 17214314 | *rs8067893* | intron variant | G | A=0.11 | 1.70E-07 | 0.08(407) | -0.23(168) | 0.03 (30) |
| 35 | 17220853 | *rs41323249* | intron variant | C | T=0.12 | 4.40E-08 | 0.07(430) | -0.12(153) | -0.73(22) |
| 36 | 17221311 | *rs41400246* | intron variant | C | T=0.10 | 3.00E-10 | 0.07(430) | -0.25(129) | -1.11(13) |
| 37 | 17226117 | *rs41525346* | intron variant | A | G=0.08 | 8.70E-07 | 0.06(512) | -0.37(88) | N.A |
| 38 | 17227704 | *rs55836267* | intron variant | T | G= 0.12 | 4.40E-08 | 0.07(430) | -0.12(153) | -0.73(22) |
| 39 | 17230965 | *rs12602831* | intron variant | G | T=0.25 | 4.10E-27 | 0.13(419) | -0.33(163) | -1.05(23) |
| 40 | 17231193 | *rs12602871* | intron variant | G | A=0.25 | 4.10E-27 | 0.13(419) | -0.33(163) | -1.05(23) |
| 41 | 17233101 | *rs76045368* | intron variant | C | T=0.25 | 4.60E-27 | 0.15(390) | -0.27(184) | -0.83(31) |
| 42 | 17233347 | *rs34518797* | intron variant | C | T=0.25 | 4.60E-27 | 0.15(390) | -0.27(184) | -0.83(31) |
| 43 | 17233992 | *rs8079562* | intron variant | G | A=0.25 | 4.50E-27 | 0.15(391) | -0.25(183) | -0.83(31) |
| 44 | 17234845 | *rs8065774* | intron variant | C | T=0.24 | 4.50E-27 | 0.15(391) | -0.25(183) | -0.83(31) |
| 45 | 17235918 | *rs41337846* | intron variant | T | C=0.24 | 5.10E-28 | 0.14(417) | -0.33(164) | -1.06(24) |

**Note:** All these SNPs were common with our dataset

**Table S9b: Alternate allele frequencies of non-coding variants of *FLCN* (found in our dataset) which are ≤ 15% in South Asian (gnomAD) and Indian (GenomeAsia100K) populations**

| **S. No.** | **COORD** | **ID** | **HGVS** | **ANNOTATION** | **REF** | **ALT** | **gnomAD (South Asian)** | **Genome Asia100K (India)** |
| --- | --- | --- | --- | --- | --- | --- | --- | --- |
| 1 | 17212251 | *rs1451192210* | *c.*1404C>T* | 3' UTR | *G* | *T* | no pop freq | no pop freq |
| 2 | 17212319 | *rs7218992* | *c.*1336G>A* | 3' UTR | *C* | *A* | 0.06 | no pop freq |
| 3 | 17213230 | *rs7224335* | *c.*425G>A* | 3' UTR | *C* | *T* | 0.05 | 0.07 |
| 4 | 17213262 | *rs12602675* | *c.*393G>A* | 3' UTR | *C* | *T* | 0.08 | 0.08 |
| 5 | 17213299 | *rs7224474* | *c.*356G>T* | 3' UTR | *C* | *A* | 0.05 | 0.07 |
| 6 | 17214195 | *rs8068606* | *c.1539-339A>C* | intron | *T* | *G* | 0.04 | 0.06 |
| 7 | 17214314 | *rs8067893* | *c.1539-458C>T* | intron | *G* | *A* | 0.11 | no pop freq |
| 8 | 17214401 | *no ID* | *c.1539-545G>A* | intron | *C* | *-* | no pop freq | no pop freq |
| 9 | 17215375 | *rs34311146* | *c.1301-59C>T* | intron | *G* | *A* | 0.08 | 0.03 |
| 10 | 17215386 | *rs544930629* | *c.1301-70C>T* | intron | *G* | *T* | no pop freq | no pop freq |
| 11 | 17215879 | *rs572265105* | *c.1300+501A>G* | intron | *T* | *C* | 0.001 | 0.0008 |
| 12 | 17219825 | *rs564603245* | *c.872-616C>T* | intron | *G* | *T* | no pop freq | no pop freq |
| 13 | 17220853 | *rs41323249* | *c.871+684G>A* | intron | *C* | *T* | 0.12 | 0.09 |
| 14 | 17221311 | *rs41400246* | *c.871+226G>A* | intron | *C* | *T* | 0.1 | 0.05 |
| 15 | 17221501 | *rs3744124* | *c.871+36G>A* | intron | *C* | *T* | 0.12 | 0.11 |
| 16 | 17222727 | *rs2292527* | *c.619-66C>T* | intron | *G* | *A* | 0.17 | 0.13 |
| 17 | 17226117 | *rs41525346* | *c.396+59T>C* | intron | *A* | *G* | 0.08 | 0.04 |
| 18 | 17226931 | *rs6502565* | *c.250-609G>A* | intron | *C* | *T* | 0.13 | 0.12 |
| 19 | 17226956 | *rs76319098* | *c.250-634G>A* | intron | *C* | *T* | 0.13 | 0.12 |
| 20 | 17227493 | *rs373903620* | *c.249+396G>A* | intron | *C* | *T* | 0.015 | 0.009 |
| 21 | 17227704 | *rs55836267* | *c.249+185A>C* | intron | *T* | *G* | 0.12 | 0.09 |
| 22 | 17228404 | *rs75336342* | *c.-24-243G>A* | intron | *C* | *T* | 0.005 | 0.003 |
| 23 | 17228589 | *rs538804082* | *c.-24-428G>T* | intron | *C* | *A* | 0.17 | 0.13 |
| 24 | 17228852 | *rs79717038* | *c.-24-691C>T* | intron | *G* | *A* | 0.13 | 0.12 |
| 25 | 17229099 | *rs565915341* | *c.-24-938C>T* | intron | *G* | *A* | 0.005 | 0.009 |
| 26 | 17234361 | *rs375429199* | *c.-227-1460C>T* | intron | *G* | *A* | 0.04 | no pop freq |
| 27 | 17236986 | *rs41345949* | *c.-302G>A* | 5' UTR | *C* | *T* | no pop freq | 0.03 |

**Table S10a: Pedigree Disequilibrium Test (PDT) for pathogenic *FLCN* mutations: pedigree informative parent triads (*X_T_*) and discordant siblines (*X_S_*) calculation to define random variable (*D*)**

| **Family ID** | **Informative Parent (*X_T_*)** | **Discordant sibships (*X_S_*)** | ***D*** |
| --- | --- | --- | --- |
| F1 | 2 (*n_t_* = 2) | 0 (*n_s_* = 1) | 0.66 |
| F2 | 0 (*n_t_* = 0) | -1 (*n_s_* = 1) | -1 |
| F4 | 0 (*n_t_* = 0) | 1 (*n_s_* = 1) | 1 |
| F5 | 3 (*n_t_* = 3) | 0 (*n_s_* = 0) | 1 |
| F11 | 1 (*n_t_* = 1) | 0 (*n_s_* = 1) | 1 |
| F12 | 2 (*n_t_* = 2) | 0 (*n_s_* = 1) | 1 |
| F13 | 0 (*n_t_* = 0) | 4 (*n_s_* = 4) | 4 |
| F15 | 1 (*n_t_* = 1) | 1 (*n_s_* = 1) | 1 |

**Table S10b: Pedigree Disequilibrium Test (PDT) for SNP *rs1708629*: Pedigree triads (*X_T_*) and siblines (*X_S_*) calculation to define random variable (*D*)**

| **Family ID** | **Informative Parent (*X_T_*)** | **Discordant sibships (*X_S_*)** | ***D*** |
| --- | --- | --- | --- |
| F2 | 0 (*n_t_* = 0) | -2 (*n_s_* = 1) | -2 |
| F4 | 0 (*n_t_* = 0) | 0 (*n_s_* = 1) | 1 |
| F7 | 8 (*n_t_* = 4) | 0 (*n_s_* = 0) | 2 |
| F8 | 1 (*n_t_* = 1) | 1 (*n_s_* = 1) | 1 |
| F13 | 0 (*n_t_* = 0) | 8 (*n_s_* = 1) | 8 |
| F15 | 1 (*n_t_* = 1) | -5 (*n_s_* = 1) | -2 |

**Table S10c: Pedigree Disequilibrium Test (PDT) for SNP *rs41345949*: Pedigree triads (*X_T_*) and siblines (*X_S_*) calculation to define random variable (*D*)**

| **Family ID** | **Informative Parent (*X_T_*)** | **Discordant sibships (*X_S_*)** | ***D*** |
| --- | --- | --- | --- |
| F4 | 0 (*n*_t_ = 0) | 1 (*n*_s_ = 1) | 1 |
| F6 | 0 (*n*_t_ = 0) | 0 (*n*_s_ = 1) | 0 |
| F7 | 8 (*n*_t_ = 4) | 0 (*n*_s_ = 0) | 2 |
| F8 | 0 (*n*_t_ = 1) | -1 (*n*_s_ = 1) | -0.5 |
| F15 | 2 (*n*_t_ = 1) | -3 (*n*_s_ = 1) | -0.5 |

**Table S11:** **Effect of *FLCN* exonic mutations on protein structure**

| **Effect of mutation** | **Exon 7 mutation** | **Exon 10 mutation** | **Exon 11 mutation** | **Exon 12 mutation** |
| --- | --- | --- | --- | --- |
| **Chromosomal position** | g.17222646 | g.17217085-17217095 | g.17216395 | g.17215284 |
| **HGVS** | c.634C>T | c.1150_1160del delGTCCAGTCAGC | c.1285delC | c.1329_1332dupAGCC |
| **Amino acid (AA) changes** | Q212* | p.V384F*2 | p.H429T*39 | p.A445S*11 |
| **Annotation** | Stop-gain | Frameshift, deletion | Frameshift, deletion | Frameshift, duplication |
| **Reference AA** | Glutamine | Phenylalanine | Histidine | Alanine |
| **Mutated AA** | Stop-codon | Valine | Threonine | Serine |
| **Protein Domain** | uDENN  (N-terminal) | cDENN (C-terminal) | cDENN  (C-terminal) | cDENN (C-terminal) |
| **CADD Score** | 43 (pathogenic) | 33 (pathogenic) | 33 (pathogenic) | 17.67 (pathogenic) |
| **Distance from Splice Site** | 16 | 17 | 16 | 33 |
| **position of stop codon in  wt/ mu CDS** | 1740/636 | 1740/1155 | 1740/1401 | 1740/1368 |
| **position of stopcodon from site of mutation** | same | 2 | 39 | 11 |
| **Stop Codon wt/mu** | 580/212 | 580/385 | 580/467 | 580/457 |
| **Loss of protein features** | 11 helices, 5 beta strands, 1 turn, coiled-coil domain | 9 helices, 4 beta strands, 1 turn | 6 helives, 2 beta strands | 5 helives, 1 beta strand |

wt: wild-type, mu: mutated

**Note:** The N-terminal or longin terminal is part of upstream/uDENN domain (*p.86* to *p.242*),
C-terminal is part of two domains - core/cDENN domain (*p.339* to *p.491*) and downstream/dDENN domain (*p.493* to *p.558*). For CADD, cut-off score of pathogenicity was decided to be 15, above which will be predicted to be pathogenic.

**Table S12: Homology modelled structural validation of protein by PROCHECK (Ramachandran plot parameters)**

| **Protein Structure (SWISSMODEL)** | **Ramachandran plot parameters (stereochemical properties)** | | | |
| --- | --- | --- | --- | --- |
|  | **Residues in core region** | **Residues in allowed region** | **Residues in disallowed region** | **Overall  G-factor** |
| FLCN (wild-type) | 80% | 17% | 0.20% | -0.28 |
| FLCN (ex7-*Q212**) | 80% | 17% | 0.60% | -0.27 |
| FLCN (ex10-*p.V384F*2*) | 79.90% | 17% | 0.30% | -0.3 |
| FLCN (ex11-*p.H429T*39*) | 82.80% | 14% | 1.10% | -0.24 |
| FLCN (ex12-*p.A445S*11*) | 86% | 11% | 0.50% | -0.27 |
| FNIP2 (wild-type) | 65% | 23% | 3.70% | -0.59 |
| RRAGA (wild-type) | 87.50% | 12.10% | 0.30% | -0.23 |
| RRAGC (wild-type) | 86% | 13.60% | 0% | -0.21 |

**Note:** PROCHECK analyses the stereochemical properties of protein structure by various parameters, with one of the key factors to be the Ramachandran plot. Residues are considered acceptable if more than 90% of the residues are in the core and overall allowed region. The G-factor measures the overall geometry, lower the G-factor, higher is a chance of something amiss in the geometry.

**Table S13a: HADDOCK Scores for wild type or four-exonic mutant FLCN docked with wild-type FNIP2, RRAGA, and RRAGC (4-protein complex)**

| **wt/mutant FLCN with wt FNIP2, RRAGA & RRAGC** | ***FLCN* mutations** | **HADDOCK score** | **Cluster size** | **RMSD from the overall lowest-energy structure** | **Van der Waals energy** | **Electrostatic energy** | **De-solvation energy** | **Restraints violation energy** | **Buried Surface Area** | **Z-Score** |
| --- | --- | --- | --- | --- | --- | --- | --- | --- | --- | --- |
| wt-FLCN | not applicable | -342.2±0.0 | 1 | 0.0±0.0 | -205.8±0.0 | -1036.0±0.0 | -30.1±0.0 | 1008.6±0.0 | 8467.7±0.0 | -2.1 |
| exon-7 mutant FLCN | *p.Q212* (c.634C>T)* | -203.2±30.5 | 2 | 25.2±3.0 | -150.7±7.3 | -746.8±39.4 | 2.5±3.2 | 944.3±120.6 | 5634.2±192.8 | 0 |
| exon-11 mutant FLCN | *p.V384F*2 (c.1150_1160del11)* | -252.1±20.6 | 9 | 37.4±3.0 | -166.8±18.1 | -930.1±151.8 | -2.5±8.0 | 1032.5±70.0 | 6639.1±331.1 | 0 |
| exon-10 mutant FLCN | *p.H429T*39 (c.1285delC)* | -264.6±18.1 | 10 | 35.6±6.0 | -178.9±17.0 | -808.9±48.8 | -14.1±8.8 | 902.7±150.5 | 6172.7±268.3 | 0 |
| exon-12 mutant FLCN | *p.A445S*11 (c.1329_1332dupAGCC)* | -242.8±2.8 | 8 | 35.2±4.5 | -163.6±10.0 | -837.7±78.8 | -13.2±10.5 | 1015.4±120.9 | 6171.2±341.2 | 0 |

Abbreviations: wt: wild-type, nuc: nucleotide

**Note:** HADDOCK is a protein-protein docking tool that docks protein monomers. Solvated docking analysis with FCC generated clusters with their corresponding desolvation energies. Z-scores indicate similarity between different clusters generated. All mutant FLCN monomers docked with
wt-FNIP2-RRAGA-RRAGC generated only one cluster each, hence the z-score was zero in those four cases. About 200 clusters were generated with greater similarity (z-score -2.1) for wt-FLCN docked with wt-FNIP2-RRAGA-RRAGC. Greater negative z-scores indicate greater similarity between the clusters. The best cluster was selected based on the HADDOCK score, their z-score, buried surface areas and RMSD values.

**Table S13b: HADDOCK Scores for wild type or four-exonic mutant FLCN docked with wild-type FNIP2 (2-protein complex)**

| **wt/mutant *FLCN* with wt-FNIP2** | **Mutated FLCN** | **HADDOCK score** | **Cluster size** | **RMSD from the overall lowest-energy structure** | **Van der Waals energy** | **Electrostatic energy** | **Desolvation energy** | **Restraints violation energy** | **Buried Surface Area** | **Z-Score** |
| --- | --- | --- | --- | --- | --- | --- | --- | --- | --- | --- |
| wt-FLCN | not applicable | -53.1±10.3 | 5 | 19.7±0.2 | -65.1±15.6 | -372.6±33.4 | -1.3±10.4 | 877.4±193.2 | 3280.5±460.2 | -1.5 |
| exon-7 mutant FLCN | *p.Q212* (c.634C>T)* | -47.9±11.9 | 7 | 20.7±0.1 | -64.5±5.8 | -130.9±21.4 | -11.7±3.0 | 544.5±49.1 | 2030.1±139.4 | -1.3 |
| exon-11 mutant FLCN | *p.V384F*2 (c.1150_1160del11)* | -32.0±19.4 | 4 | 7.1±0.3 | -49.9±6.3 | -257.9±65.5 | 0.7±4.7 | 687.8±35.9 | 2000.7±153.5 | -0.8 |
| exon-10 mutant FLCN | *p.H429T*39 (c.1285delC)* | -37.6±11.5 | 4 | 30.0±0.5 | -49.5±9.1 | -321.4±52.2 | 7.2±3.5 | 690.3±15.2 | 2201.0±90.2 | -1.5 |
| exon-12 mutant FLCN | *p.A445S*11 (c.1329_1332dupAGCC)* | -40.2±7.4 | 6 | 27.6±0.0 | -70.8±1.5 | -262.1±7.4 | 2.7±1.3 | 802.5±53.9 | 2485.0±39.3 | -1.3 |

Abbreviations: wt: wild-type, nuc: nucleotide

**Note:** Solvated docking analysis with FCC generated clusters generate their corresponding desolvation energies. Docking experiments generated
≥ 7 clusters for each complex. Z-scores indicate the structural similarity with the other clusters in the docking, the more negative z-score, and the better is the cluster. The best cluster was selected based on the HADDOCK score, their z-score, buried surface areas and RMSD values.

**Table S14: Analysis of *FLCN* copy number variation in patients, asymptomatic members and unrelated healthy controls using Exons 4, 8 and 13 Taqman copy number assay**

| **Test: Paired t-test for Patients and Asymptomatics** | | | |
| --- | --- | --- | --- |
| **Groups** | **Exon 4 (p-value)** | **Exon 8  (p-value)** | **Exon 13  (p-value)** |
| Patients vs. Asymptomatics | 0.96 | 0.7 | 0.521 |
|  | | | |
| **Test: Mann-Whitney test (Exon 4), Unpaired t-test (Exon 8 and 13)** | | | |
| **Groups** | **Exon 4 (p-value)** | **Exon 8  (p-value)** | **Exon 13  (p-value)** |
| Patients vs. Unrelated Controls | 0.825 | **0.019** | 0.186 |
| Asymptomatics vs. Unrelated controls | 0.348 | **0.008** | 0.125 |

**Note**: p-values <0.05 are given in bold. The 2^-∆ct^ values of the unrelated controls for only exon 4 assay were not in normal distribution (Kolmogorov-Smirnov tests), therefore, non-parametric Mann-Whitney tests were performed for Patients vs unrelated Controls, and Asymptomatics vs unrelated controls. Patient and asymptomatic groups were in normal distribution for all three exon assays (4, 8 and 13), therefore, parametric paired t-tests were performed between patients and asymptomatic members. Parametric unpaired t-tests were performed for exon 8 and 13 assays for patients vs unrelated controls and asymptomatic members vs unrelated controls, as unrelated controls for both assays were in normal distribution.

**Table S15: Demography and presence of *FLCN* pathogenic mutations in 27 patients suffered from PSP recurrence**

| **Family ID** | **Patient ID** | **Age-range for onset of 1^st^ PSP** | **PSP occurrences (no. of times)** | **Sex** | **Family history** | **Smoking (≥10 years)** | **Pathogenic *FLCN* mutations** |
| --- | --- | --- | --- | --- | --- | --- | --- |
| F1 | F1-1 | 31-35 | 1 | female | present | Absent | Present |
|  | F1-2 | 26-30 | 1 | female | present | Absent | Present |
| F2 | F2-9 | 41-45 | 1 | male | absent | Absent | Present |
| F3 | F3-13 | 36-40 | 3 | male | present | Absent | Present |
|  | F3-14 | 36-40 | 1 | male | present | Absent | Present |
| F4 | F4-18 | 41-45 | 2 | male | absent | Absent | Present |
| F5 | F5-25 | 31-35 | 1 | female | present | Absent | Present |
|  | F5-26 | 26-30 | 1 | male | present | Absent | Present |
|  | F5-28 | 21-25 | 2 | male | present | Absent | Present |
| F6 | F6-35 | 61-65 | 1 | male | absent | Absent | Absent |
| F7 | F7-44 | 15-20 | 4 | male | present | Absent | Absent |
|  | F7-45 | 31-35 | 1 | female | present | Absent | Absent |
|  | F7-46 | 31-35 | 1 | male | present | Absent | absent |
|  | F7-47 | 36-40 | 2 | female | present | Absent | absent |
|  | F7-48 | 55-60 | 2 | female | present | Absent | absent |
| F8 | F8-56 | 15-20 | 1 | female | present | Absent | absent |
| F10 | F10-67 | 56-60 | 1 | female | absent | Present | absent |
| F11 | F11-70 | 21-25 | 4 | female | present | Absent | present |
| F12 | F12-77 | 56-60 | 1 | female | present | Absent | present |
|  | F12-78 | 41-45 | 1 | male | present | Present | present |
| F13 | F13-82 | 46-50 | 2 | male | present | Present | present |
|  | F13-83 | 46-50 | 1 | male | present | Present | present |
|  | F13-84 | 41-45 | 1 | male | present | Present | present |
|  | F13-85 | 31-35 | 2 | male | present | Present | present |
| F14 | F14-95 | 31-35 | 1 | female | absent | Absent | present |
| F15 | F15-99 | 51-55 | 1 | male | present | Absent | present |
|  | F15-101 | 31-35 | 1 | female | present | Absent | present |

**Note:** Members of family F9 were not included in the test, since they were only presented with lung cysts. GEE (SPSS) was used for analyzing the probability of a recurrence of a PSP with age. Since, families imply clustered data, hence GEE was used for this analysis. The number of times, PSP occurrences, was taken as a dependent variable for ‘age of onset of first PSP’, while patient sex, family history, smoking habits and presence of pathogenic *FLCN* mutations were taken as co-factors. PSP occurrences: 1 (PSP has occurred once), 2 (PSP has occurred twice), 3 (PSP has occurred thrice), 4 (PSP has occurred four times). Other factors were taken as binary (as presence or absence). Pleurodesis was not taken as co-factor, as none of the patients opted for the procedure after their first PSP. Size and number of lung cysts were not considered.
