# Supplementary material for: Genetic insight into Birt–Hogg–Dubé syndrome in Indian patients reveals novel mutations at FLCN
Source: Orphanet J Rare Dis. 2022 Apr 27;17:176. doi: 10.1186/s13023-022-02326-5 (PMC9044636; doi:10.1186/s13023-022-02326-5)
Supplement: Supplementary file 4 — Additional file 4: Summary of the study [file 13023_2022_2326_MOESM4_ESM.docx]

**Summary of the study**

**Clinically Diagnosed BHDS families: 15 (F1 to F15)**
**Patients: 31, Asymptomatic family related members: 74**

| **Pathogenic FLCN variants (Protein truncating and Splice Region)** | | |
| --- | --- | --- |
| **Exon** | **Mutation** | **Family** |
| 7 | *c.634C>T* (Clinvar reported) | F5 (n=12),  Patients: 3, Asymptomatics: 2 |
| 10 | *c.1150-1160del11* (novel) | F11 (n=7),  Patients: 1, Asymptomatics: 2 |
| 11 | *c.1285delC* (reported and hotspot) | F1, F12, F13, F14, F15 (n= 37),  Patients: 11, Asymptomatics: 8 |
| 12 | *c.1332dupAGCC*  (Clinvar reported) | F4 (n=5),  Patients: 1, Asymptomatics: 0 |
| 11-12 | Splice donor*: c.1300+1G>*A (reported) | F2 (4),  Patients: 1, Asymptomatics: 2 |
| 11-12 | Splice Acceptor:  *c.1301-1G>A* (novel) | F3 (5),  Patients: 2, Asymptomatics: 2 |

**Phenotype Ontology Analysis** (n=31 patients)
Confirmed PSP/BHDS: 28 patients

***FLCN* NGS data** Read count analysis (Seqmonk).
**Regions with different depth of coverage found between patients and asymptomatics:**
exons 8-9, exons 10-14
**Families:** **4** (F3, F4, F9, F10).
(F3, F4, F9, F10)

**Taqman Copy Number Assays
(Exon 4, 8, 13)**
Patients: 7, Asymptomatics: 13. Families: 4 (F3, F4, F9, F10)
Unrelated controls: 23
**Copy number changes found - Exon 8:** Patients vs Controls
Asymptomatics vs controls

**Sanger Exonic sequencing/Validation of NGS data:**(15 families)
Patients: 31 (F1 to F15), Asymptomatics: 74 (F1 to F15)

**Targeted Amplicon NGS of *FLCN*** (11 families)
Patients: 20 (F1 to F11)
Asymptomatics: 15 (F2 to F10)

**Interesting SNPs found:**
UTR: 3 SNPs
Intronic: 6 SNPs

**Summary**

***FLCN* pathogenic Variants found in10 families**
Families: F1, F2, F3, F4, F5, F11, F12, F13, F14, F15
Patients: 19
Asymptomatics: 16

**No FLCN pathogenic variants found in 5 families**
Families: F6, F7, F8, F9, F10
Patients: 12

**Genotype vs Expression** Analysis

(eQTL data from GTex & Population datasets)
